# Pan-cancer analysis revealed H3K4me1 at bivalent promoters premarks DNA hypermethylation during tumor development and identified the regulatory role of DNA methylation in relation to histone modifications

**DOI:** 10.1186/s12864-023-09341-1

**Published:** 2023-05-04

**Authors:** Yang Lu, Qiang Cao, Yue Yu, Yazhou Sun, Xuan Jiang, Xin Li

**Affiliations:** 1 School of Medicine, Shenzhen Campus of Sun Yat-Sen University, Shenzhen, China; 2 Guangdong Provincial Key Laboratory of Digestive Cancer Research, The Seventh Affiliated Hospital of Sun Yat-Sen University, Shenzhen, China; 3 The Seventh Affiliated Hospital of Sun Yat-Sen University, Shenzhen, China

**Keywords:** Cancer, Promoter CGIs, DNA methylation, H3K4me1, LSD1, Bivalent promoters

## Abstract

**Background:**

DNA hypermethylation at promoter CpG islands (CGIs) is a hallmark of cancers and could lead to dysregulation of gene expression in the development of cancers, however, its dynamics and regulatory mechanisms remain elusive. Bivalent genes, that direct development and differentiation of stem cells, are found to be frequent targets of hypermethylation in cancers.

**Results:**

Here we performed comprehensive analysis across multiple cancer types and identified that the decrease in H3K4me1 levels coincides with DNA hypermethylation at the bivalent promoter CGIs during tumorigenesis. Removal of DNA hypermethylation leads to increment of H3K4me1 at promoter CGIs with preference for bivalent genes. Nevertheless, the alteration of H3K4me1 by overexpressing or knockout LSD1, the demethylase of H3K4, doesn’t change the level or pattern of DNA methylation. Moreover, LSD1 was found to regulate the expression of a bivalent gene *OVOL2* to promote tumorigenesis. Knockdown of *OVOL2* in LSD1 knockout HCT116 cells restored the cancer cell phenotype.

**Conclusion:**

In summary, our work identified a universal indicator that can pre-mark DNA hypermethylation in cancer cells, and dissected the interplay between H3K4me1 and DNA hypermethylation in detail. Current study also reveals a novel mechanism underlying the oncogenic role of LSD1, providing clues for cancer therapies.

**Supplementary Information:**

The online version contains supplementary material available at 10.1186/s12864-023-09341-1.

## Background

Cancer cells are featured with epigenetic changes that lead to the gene expression alterations to drive tumorigenesis and metastasis. In particular, tumor cells are characterized by global DNA hypomethylation and focal DNA hypermethylation at promoter CpG islands (CGIs) [1]. Large-scale DNA hypomethylation usually associates with genomic instability, while locus-specific hypermethylation in tumor cells usually corresponds to silenced transcription of tumor suppressor genes [2–4], for example, due to the promoter hypermethylation, *OVOL2* (ovo like zinc finger 2), *MLH1* and *CDKN2A* (*p16/ARF*) were silenced in colorectal tumors [5–7], *BRCA1* was silenced in ovarian cancer [8] and breast cancer [9]. Bivalent genes are a subset of genes originally identified in embryonic stem cells (ESCs), and play key roles in development [10, 11]. Promoters of bivalent genes are frequent targets of DNA hypermethylation in human cancers [12, 13]. The acquisition of DNA hypermethylation blocks the expression of these bivalent genes and pushes cells into an ESCs state with strong self-renewal and multi-directional differentiation ability [2, 14–16]. The promoter DNA hypermethylation in cancer is generally universal and has been found in a variety of cancer types, however, some of the promoter DNA hypermethylation is tumor-type specific, probably due to the tissue of origin specificity of tumors [17, 18].

In addition to DNA methylation, histone modification is another well-studied epigenetic regulation that plays a significant role in cancer development [19]. Evidence suggest that DNA hypermethylation and histone modification can be dependent on each other and cooperate to orchestrate gene expression during development and tumorigenesis [19–21]. The promoters of bivalent genes are marked by histone modifications, including the repressive mark H3K27me3 and the active mark H3K4me3, to prepare these key developmental genes either for rapid activation upon differentiation signals, or remaining in the repressive state without further signals [10, 22]. The bivalent genes identified in ESCs highly overlap with the genes whose promoter CpG is hypermethylated in cancers [10, 15]. Typically, bivalent promoters in normal cells are hypomethylated [23], however, a number of seminal papers reported that in tumor cells bivalent promoters gain DNA methylation while losing the histone modifications in the process of cancer development [15, 24–26]. Moreover, the DNA hypermethylation of bivalent promoters in cancers closely correlates with the H3K27me3/H3K4me3 ratio of that in embryonic stem cells [25].

The histone modification H3K4me1 is usually enriched at enhancers, however, recently, increased evidence indicate that H3K4me1 also exists at promoters to regulate gene transcription [27–30]. H3K4me1 are distributed in two alternative models around promoters, either unimodal pattern at poised promoters or bimodal pattern at active promoters [31]. In mammals, H3K4 is methylated by COMPASS (Complex of Proteins Associated with Set1, COMPASS) complex [30] and demethylated by histone demethylase (HDM) LSD1 or LSD2 [32]. LSD1 was found to mostly bind with promoters, and manipulation of LSD1 can efficiently alter the enrichment of H3K4me1 at promoter regions [33, 34]. Similar to the histone modification H3K27me3 and H3K4me3, which correlates with DNA hypermethylation at bivalent promoters in cancer cells, the pattern of H3K4me1 is also reported to associate with DNA hypermethylation pattern in the certain type of cells: the distribution of H3K4me1 in hESCs and normal prostate epithelial cells (PrECs) could pre-mark the DNA hypermethylation encroachment around CGIs in prostate cancer cells (LNCaP); disruption of H3K4me1 by inactivating MLL3/MLL4, the key components of COMPASS complex, alters the DNA methylation pattern at promoter CGIs in mESCs and mouse primary B cells [35]. However, the correlation is not studied in other cell types. Based on this evidence, we sought to systematically explore whether H3K4me1 pattern at promoter CGIs in tissue normal counterparts pre-marks DNA hypermethylation during carcinogenesis. Furthermore, we also aimed to explore the reciprocal regulatory interaction between DNA methylation and H3K4me1.

LSD1 has been demonstrated to promote the growth and metastasis of a variety of tumor cells [36–40]. The up-regulation of LSD1 correlates with advanced cancer stage, high cancer grade and poor prognosis [41]. In addition to demethylating histones, LSD1 can also demethylate non-histone proteins, moreover, LSD1 targets nonhistone proteins to perform multiple demethylase-independent functions, such as protein degradation, cell autophagy, and so on [40]. Due to the pleiotropic role of LSD1, the mechanism of LSD1 as an oncogene is complicated and highly diverse in different types of cancers.

In this study, we revealed that the decrease in H3K4me1 levels coincides with DNA hypermethylation at the bivalent promoter CGIs in the development of various types of cancers. Therefore, DNA hypermethylation accrual at tumor promoter CGIs can be predicted by H3K4 monomethylation pattern of their tumor-origin tissues. Furthermore, alterations of DNA methylation at bivalent promoter CGIs dramatically affect the distribution pattern of H3K4me1, but the change of H3K4me1 through overexpressing or knockout LSD1 had no effect on the DNA methylation pattern. In addition, we provided evidence that LSD1 plays an oncogenic role in colon cancer through epigenetic regulation. Mechanistically, knockout of LSD1 upregulates the transcription of bivalent genes, such as *OVOL2*, via modifying the H3K4 methylation at promoter regions to inhibit the growth and metastasis of cancer cells. Our study revealed the mechanisms of epigenetic regulation of LSD1 and shed light on the therapeutic potential of LSD1.

## Results

### Characterization of genes with hypermethylated promoters in different types of cancer

**Fig. 1:**
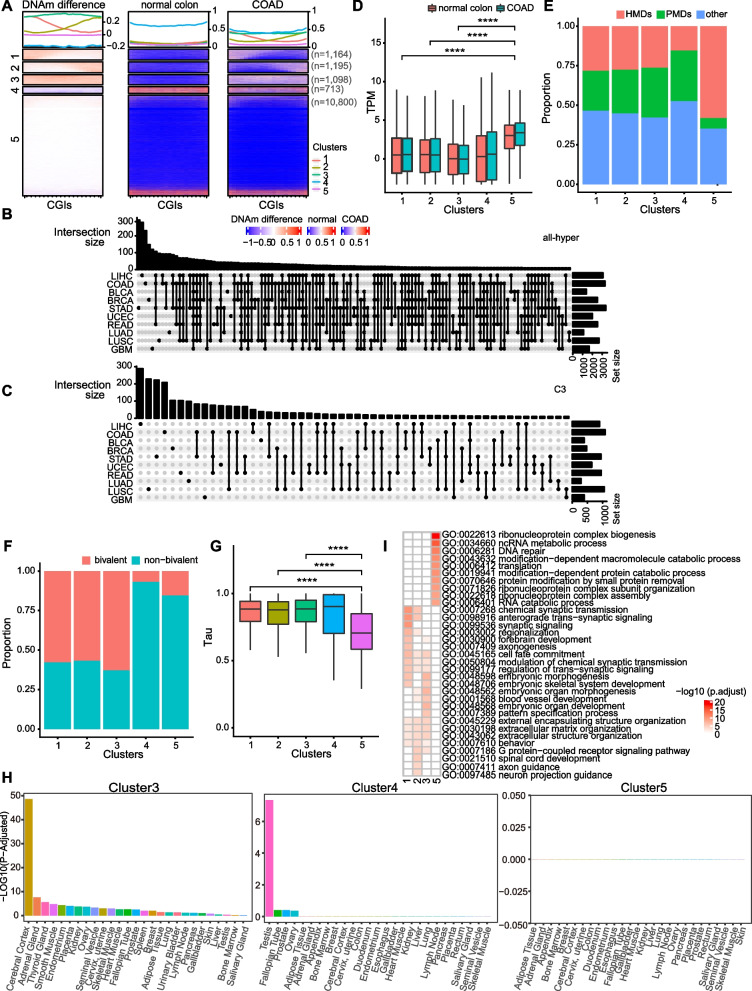
Characterization of different group genes with promoter CGIs in COAD. **A** Left panel shows the DNA methylation difference of COAD over normal colon tissue. Middle and right panels show DNA methylation patterns at promoter CGIs of normal colon tissue and COAD specifically. Each line represents a single CGI. **B** Upset plot showing the overlap of all-hyper group genes in different cancer types. **C** Upset plot showing the overlap of C3 group genes in different cancer types. **D** The box plot showing the abundance of genes from 5 groups in normal colon tissue or COAD. TPM: transcripts per million. Data were presented as mean ± SD. Statistical analysis was performed by Wilcoxon Rank Sum, ****p < 0.0001. **E** The stacked bar plot showing the distribution of promoter CGIs in 5 groups in PMDs, HMDs and other regions in COAD. PMDs: partially methylated domains, HMDs: highly methylated domains. **F** The stacked bar plot showing proportion of bivalent and non-bivalent genes in 5 groups in COAD. **G** The box plot showing Tau index of genes in 5 groups in COAD. Data were presented as mean ± SD. Statistical analysis was performed by Wilcoxon Rank Sum, **** p < 0.0001. **H** Tissue enrichment of genes in C3, C4 and C5 groups in COAD. **I** Heat map showing GO analysis of genes involved in different groups in COAD

Alteration of promoter DNA methylation is an epigenetic hallmark of cancer cells. To systematically explore the biological significance of the aberrant DNA methylation pattern in different cancer types, whole-genome bisulfite sequencing (WGBS) data of 10 different tumors including bladder urothelial carcinoma (BLCA), breast invasive carcinoma (BRCA), colon adenocarcinoma (COAD), lung adenocarcinoma (LUAD), lung squamous cell carcinoma (LUSC), rectum adenocarcinoma (READ), stomach adenocarcinoma (STAD), uterine corpus endometrial carcinoma (UCEC), glioblastoma multiforme (GBM), liver hepatocellular carcinoma (LIHC), and tissue normal counterparts, were collected from previous publications [42, 43] and subjected to analysis. In each type of cancer, k-means clustering analysis classified all genes into 5 groups based on the DNA methylation difference of their promoter CGIs between tumors and tissue normal counterparts (Fig. 1A, Supplementary Fig. S1, S2A, and Supplementary Table S1). Genes in C1 group showed 5’ DNA hypermethylation accrual at promoter CGIs in tumor tissues, while genes in C2 group showed 3’ DNA hypermethylation accrual in tumor tissues. The promoters of C3 genes showed hypermethylation across the entire CGIs in tumor tissues compared with normal control tissues. C1, C2 and C3 groups with CGI hypermethylation were collectively named as “all-hyper” group. Promoters of C4 genes showed hypomethylation in tumor tissues, while genes in C5 group showed comparable promoter DNA methylation level between tumor and tissue normal counterparts. The C4 group contains the least number of genes in all cancer types but BLCA, and C5 group had the largest number of genes in each type of cancer. (Fig. 1A, Supplementary Fig. S1, S2A). Next, we evaluated the cancer-type-specificity of the above classification by overlapping genes among each group of different cancers. Results showed quite a lot overlap among genes in the all-hyper group of different cancers (Fig. 1B), however, genes in C3 and C4 group of different cancers showed minimal overlap (Fig. 1C and Supplementary Fig. S2B). These results indicate that a substantial number of genes’ promoter CGIs were prone to hypermethylation across all the cancer types, however, the methylation susceptibility, accrual mode and degree of hypermethylation have large variations among different cancer types.

To further characterize the five groups of genes, we analyzed the expression level, tissue specificity and distribution of the 5 group genes along the genome. Generally, genes of all-hyper group are expressed at a lower level compared with genes in C5 groups (Fig. 1D, Supplementary Fig. S3A). Global hypomethylation is a feature of cancer genome, and the partially methylated domains (PMDs) are not as stable as other parts of genome due to the high chance of being inserted by transposable elements [42, 44]. Large regions that are highly methylated are termed as highly methylated domains (HMDs), albeit some local regions such as promoter CGIs and other regulatory elements within HMDs are unmethylated. HMDs were usually associated with active chromatin status and high transcription [45]. Significantly higher percentage of genes in all-hyper group (~ 30%) are distributed in PMDs, in contrast with 6% of C5 genes distributed in PMDs (Fig. 1E). PMDs are usually at megabase-scale with low gene density and poor transcription activity [45]. Consistently, genes in all-hyper group are distributed more in PMDs and expressed at a lower level compared with genes in C5 group (Fig. 1D, E, Supplementary Fig. S3A, B). We next evaluated the overlap of these genes with bivalent genes. The human bivalent genes list was obtained from a previous study, which identified high-confidence bivalent genes based on several publicly available ChIP-seq datasets from hESCs [46]. Results showed that about 59.07% of genes in C1- C3 groups in COAD were bivalent genes, however, only 15.47% genes in C5 group were bivalent genes (χ^2^ test, *p*-value < 2.2e-16, Fig. 1F). All the other types of cancers show similar characteristics: significantly more genes in all-hyper group are bivalent genes compared with genes in C5 group (Supplementary Fig. S3C). This is consistent with the previous observation that bivalent promoters tend to be hypermethylated in cancers [13, 15]. In addition, we also analyzed the tissue-specificity of above 5 groups genes. Tau index calculation using the expression data from the Genotype-Tissue Expression project (GTEx) was applied to evaluate tissue specificity of genes in each group. Tau index close to 1 indicates expression in one tissue (high tissue-specificity), while Tau index close to 0 indicates uniform expression across all the tissues. As shown in Fig. 1G, genes in all-hyper group in COAD show high tissue-specificity, while genes in C5 group bear significantly lower tissue-specificity. Analysis in other cancers showed similar pattern (Supplementary Fig. S3D). Gene expression data from another database Human Protein Atlas (HPA) [47] was used to confirm the tissue specificity of genes in each group. Lots of tissue-specific genes were significantly enriched in C3 group while there is no enrichment of tissue-specific genes in C5 groups (Fig. 1H). To elucidate the biological function of each group, Gene Ontology (GO) enrichment analysis was performed with genes of all groups in different types of cancers. Results showed that genes of all-hyper group in COAD were more enriched in the biological processes that related to differentiation and development, while the C5 cluster genes were mainly enriched in the macromolecule and protein catabolic processes, ribonucleoprotein complex subunit organization and protein translation (Fig. 1I). Moreover, this enrichment pattern is observed in all the other cancer types as well (Supplementary Fig. S4). Taken together, the genes that are hypermethylated in cancer (C1-3 group) tend to be lowly expressed and distributed in PMDs. Consistently, these genes are mainly bivalent genes with high tissue expression specificity, maintained in a transcriptionally repressed state and are more involved in pathways related to cell differentiation and tissue development [15, 48–51].

### The decrease in H3K4me1 levels coincides with DNA hypermethylation at the promoter CGIs in the process of cancer development

**Fig. 2:**
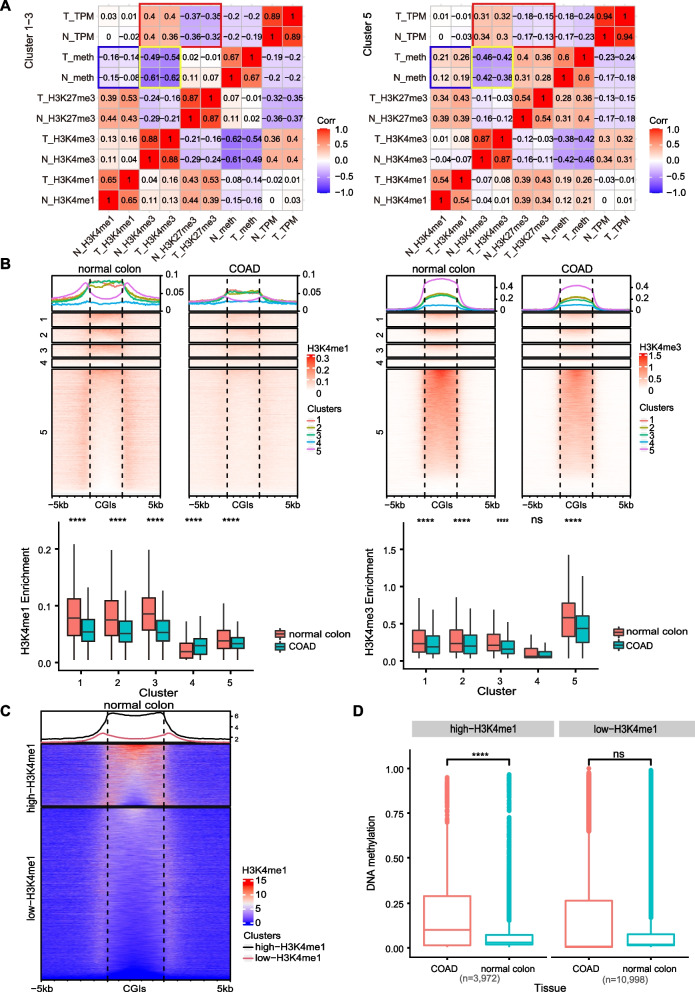
Correlation between H3K4me1 and DNA hypermethylation at promoter CGIs of cancer cells. **A** Heatmaps showing pairwise spearman correlation coefficients among multiple epigenetic modifications and gene expression. N, normal colon tissue. T, COAD. TPM: transcripts per million. **B** Up panels show the level and pattern of H3K4me1 (left) and H3K4me3 (right) at promoter CGIs and flanked regions in normal colon tissue and COAD. Lower panels show quantification of the levels of H3K4me1 at promoter CGIs of 5 group genes in normal colon tissue and COAD. Data were presented as mean ± SD. Statistical analysis was performed by Wilcoxon Rank Sum, ns: not significantly, **** p < 0.0001. **C** Distribution pattern and level of H3K4me1 at promoter CGIs and flanked regions in high-H3K4me1 group and low-H3K4me1 group. **D** Box plot showing DNA methylation level at promoter CGIs in normal colon tissue and COAD. Data were presented as mean ± SD. Statistical analysis was performed by Wilcoxon Rank Sum, ns: not significantly, **** p < 0.0001

As elaborated above, DNA methylation and histone methylation correlates with each other [19, 52], the DNA methylation and multiple histone modifications level of promoter CGIs of genes in C1-C5 groups were analyzed and the correlations among DNA methylation, histone methylation and gene expression were calculated. As expected, the repressive histone modification H3K27me3 negatively correlates with gene expression and the active histone modification H3K4me3 positively correlates with it (red box in Fig. 2A). Consistent with the previous report that H3K4me3 inhibits DNA methylation by preventing the binding of DNA methyltransferase [19, 53], the H3K4me3 are negatively correlated with DNA methylation level in both all-hyper group and C5 group (yellow box in Fig. 2A). Intriguingly, H3K4me1 negatively associates with DNA methylation in all-hyper group, while positively correlates with DNA methylation in C5 group (blue box in Fig. 2A). Analysis in other tissues showed similar pattern (Supplementary Fig. S5). The distribution patterns of H3K4me1 and H3K4me3 around promoter CGIs in cancers and tissue normal counterparts were further compared. The H3K4me3 levels of all the five groups are comparable between cancers and tissue normal counterparts (Fig. 2B). Moreover, the H3K4me3 distribution patterns of all groups are also the same, typical unimodal patterns in both normal tissue and cancer (right panel in Fig. 2B). However, the pattern and enrichment level of H3K4me1 are quite different among five groups between normal and cancer tissues: the all-hyper group showed unimodal pattern (Fig. 2B, orange, chartreuse, green lines in left panel) while the C5 group showed a bimodal pattern in both normal tissue and cancers (Fig. 2B, pink lines in left panel); the levels of H3K4me1 in promoter CGIs in all-hyper group are significantly higher in tissue normal counterparts compared with cancers (Fig. 2B, orange, chartreuse, green lines in left panel), while the level of H3K4me1 at promoter CGIs in C5 group is comparable between cancers and tissue normal counterparts (Fig. 2B, pink lines in left panel). In contrast to the decreased H3K4me1 level at promoter CGIs of all-hyper group, the level of DNA methylation of these promoter CGIs significantly increased in cancer cells as previously shown (Fig. 1A). These results indicate that the decrease in H3K4me1 levels coincides with DNA hypermethylation at promoter CGIs in the process of cancer development. Moreover, the distribution pattern and level of H3K4me1 of promoter CGIs in normal tissue can be evaluated as an indicator for DNA hypermethylation during tumorigenesis. Consistently, previous studies showed that the pattern of H3K4me1 at CpG island borders of PrECs correlates with the mode of encroachment of DNA hypermethylation in the prostate cancer cell line LNCaP cells [35]. To further confirm this observation, all promoter CGIs were classified into two groups based on their H3K4me1 enrichment in normal tissues (Fig. 2C, Supplementary Fig. S6A). The DNA methylation levels of these two groups were evaluated in both cancers and tissue normal counterparts. As expected, the DNA methylation level of the H3K4me1-high group is significantly higher in cancers compared with tissue normal counterparts, while the H3K4me1-low group showed no significant difference in DNA methylation level between cancers and tissue normal counterparts (Fig. 2D, Supplementary Fig. S6B). Taken together, the level of H3K4me1 at promoter CGIs in normal tissues pre-marks the CGIs those are prone to DNA hypermethylation during tumor development.

### Alterations in DNA methylation at bivalent promoter CGIs could affect the enrichment of H3K4me1

**Fig. 3:**
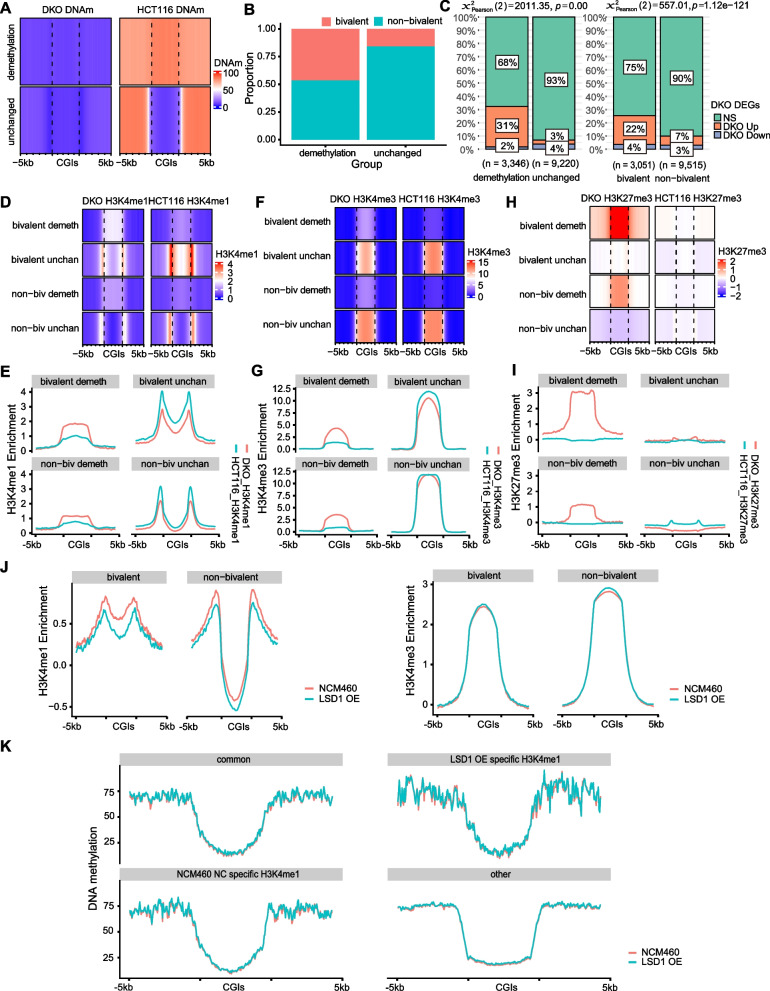
DNA demethylation at promoter CGIs alters the pattern and level of histone modification. **A** Heatmaps showing DNA methylation profiles at promoter CGIs and flanked regions of demethylation group genes and unchanged group genes in DKO cells (left panels) and WT HCT116 cells (right panels). DNAm, DNA methylation. **B** The stacked bar plot showing proportion of bivalent and non-bivalent genes in demethylation group and unchanged group. **C** The stacked bar plot showing proportion of up-regulated (DKO Up), down-regulated (DKO Down) and not significantly (NS) changed genes in demethylation and unchanged groups (left panels), as well as in bivalent and non-bivalent genes groups (right panels). **D**, **F**, **H** Heatmaps showing enrichment of H3K4me1, H3K4me3 and H3K27me3 at promoter CGIs and flanked regions of each group of genes in WT and DKO HCT116 cells. **E**, **G**, **I** Quantification of D, F, H by average plots. (**D**-**I** bivalent demeth: group of bivalent genes with demethylated promoter CGIs; non-biv demeth: group of non-bivalent genes with demethylated promoter CGIs; bivalent unchan: group of bivalent genes with unchanged promoter CGIs; non-biv unchan: group of non-bivalent genes with unchanged promoter CGIs.) **J** Average plot showing the distribution pattern and level of H3K4me1 (left) and H3K4me3 (right) at promoter CGIs and flanked regions of bivalent and non-bivalent genes in NCM460 and LSD1 OE cells. **K** Average plot showing the distribution pattern and level of DNA methylation at promoter CGIs and flanked regions of 4 groups of genes in NCM460 and LSD1 OE cells. common: group of genes with H3K4me1 peaks in both NCM460 and LSD1 OE cells (*n* = 1,011); LSD1 OE specific H3K4me1: group of genes with H3K4me1 peaks only in LSD1 OE cells (*n* = 233); NCM 460 specific H3K4me1: group of genes with H3K4me1 peaks only in NCM 460 cells (*n* = 1,141); other: other genes (*n* = 12,585).

Since enrichment of H3K4me1 at promoter CGIs in tissue normal counterparts correlates with the DNA hypermethylation in cancers, the interplay between DNA methylation and H3K4me1 is further evaluated. WGBS data, H3K4me1/3 and H3K27me3 ChIP-seq data of HCT116 cells lacking two DNA methyltransferases–DNMT1 and DNMT3B (DKO) were downloaded from the previous study [54] and subjected to re-analysis. As expected, DKO HCT116 cells showed global demethylation compared to wild type (WT) HCT116 (Fig. 3A). All promoter CGIs were classified into two groups based on the DNA methylation difference between WT and DKO HCT116 cells: 4,549 promoter CGIs that are hypermethylated in WT HCT116 cells and demethylated in DKO cells were defined as demethylation group; 10,421 promoter CGIs that are unmethylated in both WT and DKO cells were defined as unchanged group (Fig. 3A). We next evaluated the percentage of bivalent genes in the two groups: about 50% of genes in demethylation group were bivalent genes while only about 12.5% of genes in unchanged group were bivalent (χ^2^ test, *p*-value < 2.2e-16, Fig. 3B). The DNA methylation changes at promoter CGIs showed strong negative correlation with gene expression changes between WT and DKO cells (Spearman correlation coefficient: -0.43). Furthermore, the demethylation of genes in the demethylation group in DKO cells resulted in higher percentage of upregulated genes compared with genes in the unchanged group (Fig. 3C, left panel, 31% vs 3%). The demethylation in DKO cells have higher influence on gene expression for bivalent genes compared to non-bivalent genes: about 22% of bivalent genes were upregulated, while only about 7% of non-bivalent genes were increased in DKO cells compared to WT cells (Fig. 3C, right panel). ChIP-seq data analysis showed that H3K4me1/3 and H3K27me3 levels at the promoter CGIs of demethylation group genes significantly increased upon double DNMTs KO compared with WT cells (Fig. 3D, E left 2 panels, F, G left 2 panels, H, I left 2 panels), while the H3K4me1/3 and H3K27me3 levels at the promoter CGIs of unchanged group genes are comparable between DKO and WT cells (Fig. 3D, E right 2 panels, F, G right 2 panels, H, I right 2 panels). Furthermore, the bivalent genes in demethylation group showed more increase of H3K4me1 and H3K27me3 compared with non-bivalent genes (Fig. 3D, E left 2 panels, H, I left 2 panels), while the increase of H3K4me3 in demethylation group are comparable between bivalent and non-bivalent groups (Fig. 3F, G left 2 panels). These data indicated that the dynamics of DNA methylation impacts not only the gene expression, but also the level of histone modification, especially at bivalent promoter CGIs.

**Fig. 4:**
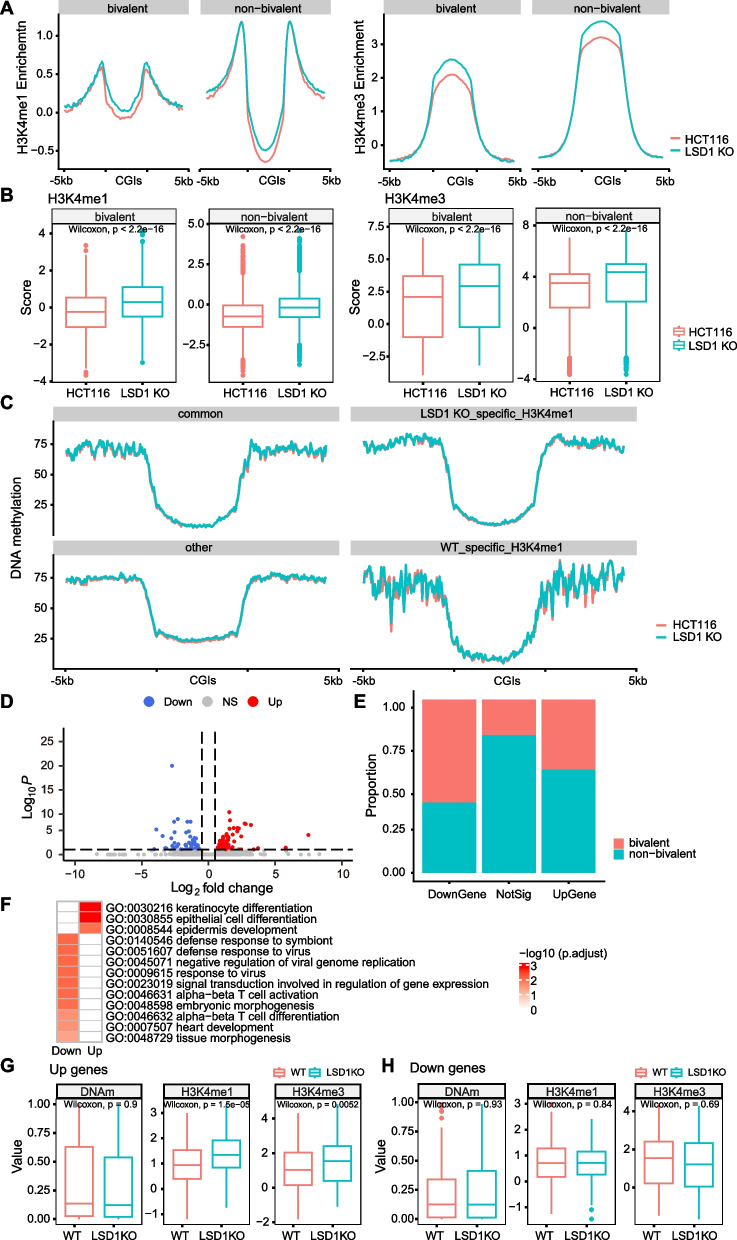
LSD1 knockout led to increased H3K4me1 at promoter CGIs. **A** Average plot showing the distribution pattern and level of H3K4me1 (left) and H3K4me3 (right) at promoter CGIs and flanked regions of bivalent and non-bivalent genes in HCT116 and LSD1 KO cells. **B** Box plots showing levels of H3K4me1 (left) and H3K4me3 (right) at promoter CGIs of bivalent and non-bivalent genes in WT and LSD1 KO HCT116 cells. Data were presented as mean ± SD. Statistical analysis was performed by Wilcoxon Rank Sum. **C** Average plot showing the distribution pattern and level of DNA methylation at promoter CGIs and flanked regions of 4 groups of genes in WT and LSD1 KO HCT116 cells. common: group of genes with H3K4me1 peaks in both WT and LSD1 KO cells (*n* = 1,669); LSD1 KO-specific-H3K4me1: group of genes with H3K4me1 peaks only in LSD1 KO cells (*n* = 3,040); WT-specific-H3K4me1: group of genes with H3K4me1 peaks only in WT cells (*n* = 364); other: other genes (*n* = 9,897). **D** Volcano plot showing DEGs between WT and LSD1 KO HCT116 cells. Up: genes that are upregulated in LSD1 KO cells compared with WT cells (*n* = 96); Down: genes that are downregulated in LSD1 KO cells compared with WT cells (*n* = 61). *q* value < 0.1 was set as a cutoff for DEGs. **E** The stacked bar plot showing the percentage of bivalent and non-bivalent genes in different groups of DEGs. **F** GO analysis of genes that were up- and down-regulated in LSD1 KO cells compared with WT cells. **G**, **H** Box plots showing levels of DNA methylation (DNAm), H3K4me1 and H3K4me3 level at promoters of Up genes and Down genes in WT and LSD1 KO cells. Up genes, genes that are upregulated in LSD1 KO cells compared with WT cells. Down genes, genes that are downregulated in LSD1 KO cells compared with WT cells. Data were presented as mean ± SD. Statistical analysis was performed by Wilcoxon Rank Sum

We further evaluated whether alteration of H3K4me1 influence the DNA methylation at promoter CGIs vice versa. LSD1, the demethylase of H3K4, was overexpressed in NCM460 cell line (LSD1 OE) or ablated in HCT116 cells to manipulate the methylation of H3K4. First, LSD1 was successfully overexpressed (Supplementary Fig. S7A, B), and caused significant decrease of H3K4me1 on both bivalent and non-bivalent promoter CGIs, the decrease of H3K4me1 on bivalent promoter CGIs was more significant than that on non-bivalent promoter CGIs (Wilcoxon Rank Sum, *p*-value < 8.8e-9, Fig. 3J, left panel), although the H3K4me3 level was not altered (Fig. 3J, right panel) in LSD1 OE cells compared with WT cells. Consistently, the peaks number of H3K4me1 reduced from 72,687 (in WT cells) to 44,397 (in LSD1 OE cells), 32,970 H3K4me1 peaks were identified exclusively in control cells, while only 4,753 peaks were identified specifically in LSD1 OE cells (Supplementary Fig. S7C). The distributions of all these H3K4me1 peaks were further analyzed and results showed that all these four groups of H3K4me1 peaks were distributed in a similar pattern (Supplementary Fig. S7D). To examine whether the change of H3K4me1 affects DNA methylation level, reduced representation bisulfite sequencing (RRBS) was performed on NC and LSD1 OE cells. All the promoters were classified into four groups based on the distribution of H3K4me1 peaks in NC or LSD1 OE cells: promoters with H3K4me1 in both NC and LSD1 OE cells (common), promoters with H3K4me1 in only LSD1 OE cells, promoters with H3K4me1 in only NC cells, and other promoters. Intriguingly, DNA methylation levels are not changed in any of these four groups (Fig. 3K). Next, the LSD1 was successfully ablated with CRISPR/Cas9 system as shown in Supplementary Fig. S8A. As expected, the H3K4me1/3 levels were significantly increased at both bivalent and non-bivalent promoter CGIs regions in LSD1 KO cells compared with WT (Fig. 4A, B). The H3K27me3 was not enriched at promoter CGIs in both WT and LSD1 KO cells, as shown in Supplementary Fig. S8B that the enrichment value is below 0. The distributions of H3K4me1 and H3K4me3 are not changed (Supplementary Fig. S9A). RRBS results showed that the DNA methylation at promoter CGIs was not altered in LSD1 KO cells although the H3K4me1/3 levels are increased (Fig. 4C).

Taken together, the dynamics of DNA methylation affect the histone modifications, especially H3K4me1 at bivalent promoters. However, alterations of H3K4me1 at promoter CGIs don’t affect the DNA methylation, indicating that dynamics of DNA methylation impose changes to histone modifications, but not vice versa.

### LSD1 preferentially regulates bivalent promoters

As shown above that the DNA methylation is not altered upon change of H3K4me1, we next studied the transcriptome dynamics upon the H3K4me1 alteration. Only 157 differentially expressed genes (DEGs), including 96 up- and 61 down-regulated genes, were identified in LSD1 KO cells (Fig. 4D). These DEGs had a larger proportion of bivalent genes compared with genes that are not changed in LSD1 KO cells (Fig. 4E). GO analysis showed that DEGs were enriched in developmental and differentiation terms (Fig. 4F), consistent with the biological relevance of bivalent genes.

To further elucidate the mechanism of the differential gene expression caused by LSD1 KO, H3K4me1 and H3K4me3 levels at promoters (TSS ± 2 kb) of these genes were analyzed. The upregulated genes showed significantly increased H3K4me1/3 at promoters, while the downregulated genes showed similar level of H3K4me1/3 (Fig. 4G, H, Supplementary Fig. S9B, C). This result indicates that the genes upregulation in LSD KO cells may be due to the enrichment change of histone modification H3K4me1/3 at promoter CGIs, but the genes downregulations are not.

### LSD1 regulates cancer cells growth and metastasis through regulating bivalent gene expression

**Fig. 5:**
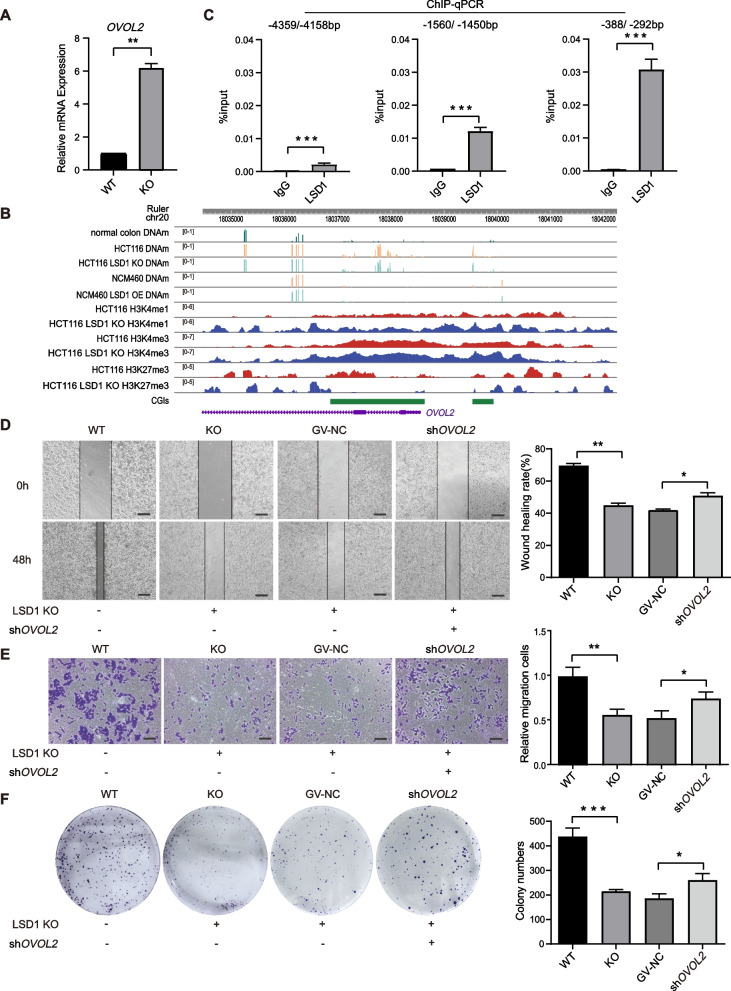
LSD1 regulates the proliferation and migration of HCT116 cells via *OVOL2*. **A** qPCR results showing the *OVOL2* gene expression level in WT and LSD1 KO HCT116 cells. The experiments performed with three biological replicates. Data were presented as mean ± SD. Statistical analysis was performed by Student’s *t* test, **p < 0.01. **B** Integrative Genomics Viewer (IGV) browser track depicting distribution pattern and levels of DNA methylation at *OVOL2* promoter in normal colon tissue, WT and LSD1 KO HCT116 cells, as well as control and LSD1 OE NCM460 cells (top 5 panels); distribution pattern and levels of H3K4me1/3 and H3K27me3 at *OVOL2* promoter in WT and LSD1 KO HCT116 cells (lower 6 panels); CGIs were highlighted by green bars, DNAm: DNA methylation. **C** ChIP-qPCR of LSD1 at *OVOL2* promoter and a control region in HCT116 cells. TSS was calculated as 0. IgG was used as a control for LSD1 antibody. control region: -4359/-4158 bp. The experiments performed with three biological replicates. Data were presented as mean ± SD. Statistical analysis was performed by Student’s *t* test, ***p < 0.001. **D** Wound healing assay results showed the migration of indicated cells at 0 h and 48 h (left panel). Quantification of the images was plotted in the right panel. Scale bar, 100 µm. The experiments were performed with three biological replicates. Data were presented as mean ± SD. Statistical analysis was performed by Student’s *t* test, ns: not significantly, * p < 0.05, ** p < 0.01. **E** Left panels show the representative images of transwell assay of indicated cells. Scale bar, 100 µm. Quantification of the images was plotted in the right panel. The experiments were performed with three biological replicates. Data were presented as mean ± SD. Statistical analysis was performed by Student’s *t* test, * p < 0.05, ** p < 0.01. **F** Left panels show the representative images of colony formation assay of the indicated cells. Quantification of the images was plotted in the right panel. The experiments were performed with three biological replicates. Data were presented as mean ± SD. Statistical analysis was performed by Student’s *t* test, * p < 0.05, *** p < 0.001

Multiple previous studies showed that LSD1 promotes tumor development in colon cancer, however, the mechanisms are not fully studied [37, 55, 56]. In particular, some studies conflict with each other: high LSD1 in colon cancer tissues mediated alteration of epigenetic modifications which contributes to the progress and metastasis of cancer cells [57], however, Jin et el. reported that global change of histone modifications were not involved in the tumorigenicity of LSD1 in HCT116 cells [55]. To elucidate the oncogenic mechanisms of LSD1, the gene expression profiling, ChIP-seq and DNA methylation profiling data of LSD1 KO cells were combined and analyzed for the potential target via which LSD1 mediated the tumorigenesis. *OVOL2* is a bivalent gene (Supplementary Fig. S10) and a colorectal tumor suppressor gene which inhibits the colorectal tumor progression and metastasis [5]. It was identified to be significantly upregulated in LSD1 KO cells compared with WT cells (Fig. 5A), and down-regulated in LSD1 OE HCT116 cells compared to NC cells (Supplementary Fig. S11A). The promoter CGI of *OVOL2* was hypermethylated in colon cancer cells HCT116 compared with normal colon tissue (Fig. 5B), and the level of H3K4me1/3 at promoter CGIs of *OVOL2* was significantly increased in LSD1 KO cells, the DNA methylation level at promoter CGI of *OVOL2* was comparable between control and LSD1 KO or LSD1 OE cells (Fig. 5B). To find out whether the promoter of *OVOL2* is a direct target of LSD1, ChIP-qPCR was performed. Results showed that LSD1 specifically binds to a distal promoter region 1.5 kb upstream to the transcription start site (TSS) and a proximal promoter region 330 bp upstream to the TSS (Fig. 5C).

We next performed wound healing and transwell assays to evaluate the migration ability of the four groups of cells: WT, LSD1-KO, mock (LSD1-KO + GV-NC) and LSD1-KO + *OVOL2* knockdown (Fig. 5D, E and Supplementary Fig. S11B). Consistent with the previous reports [58–60], LSD1-KO cells showed decreased cell migration ability, moreover, knockdown *OVOL2* in LSD1-KO cells successfully restored cell migration (Fig. 5D, E). Colony formation assay was performed to evaluate the proliferation features of these four groups of cells. Loss of LSD1 greatly inhibited the colony formation abilities of HCT116 cells, indicating decreased proliferation in LSD1-KO cells. Knockdown *OVOL2* in LSD1-KO cells successfully rescued this phenotype (Fig. 5F). Taken together, these data indicate that *OVOL2* mediates the decrease of cell proliferation and migration in LSD1 KO cells. This effect was independent of DNA methylation at the *OVOL2* promoter CGI.

## Discussion

Aberrant DNA hypermethylation happens on the promoter CGIs during the development of many types of cancers [61, 62]. Alterations of histone modifications around CGIs also occur at various kinds of tumors. However, the relationship between DNA methylation and histone modification is still obscure. Here, we performed pan-cancer analysis of aberrant DNA methylation at promoter CGIs and explored the relationship between CGIs DNA hypermethylation and histone modification changes. It is found that the decrease in H3K4me1 levels coincides with DNA hypermethylation at the bivalent promoter CGIs in cancer cells, so the H3K4me1 in normal tissue could pre-mark the DNA hypermethylation in the corresponding tumor. Alteration in DNA methylation at bivalent promoter CGIs led to changes of H3K4me1, however, dynamics of H3K4me1 seems to not impact the global DNA methylation pattern, although we couldn’t exclude the possibility that some specific loci could undergo the change of DNA methylation upon global change of H3K4me1. Furthermore, LSD1, the demethylase of H3K4me1, promotes cancer cell proliferation and migration through inhibiting the expression of *OVOL2* via regulating the histone modification level of its promoter CGI. Our study systemically established H3K4me1 as a pre-mark for DNA hypermethylation at promoter CGIs across multiple cancer types and dissected the interplay between H3K4me1 and DNA hypermethylation. In particular, a novel molecular mechanism of LSD1 mediated tumor development was identified, deepening the understanding of LSD1’s role in regulating epigenetic modification, gene expression and tumorigenesis.

DNA hypermethylation at promoters was a general epigenetic feature during tumorigenesis [20, 63], moreover, previous studies also found the aberrant DNA methylation at promoter CGIs was highly cancer-type specific [50]. Consistently, the genes of C3 group (full-hypermethylation group) didn’t show much overlap among different types of cancers. Previous studies also indicated that hypermethylated genes in breast cancers had tissue-specific expression patterns [49]. Our results showed that hypermethylated genes in all the other cancer types are also prone to be expressed in a tissue-specific pattern. Our study extended the previous occasional observations to a broader spectrum of cancers, which deepens the understanding of epigenetic dynamics in tumors.

DNA hypermethylation at promoter CGIs results in repressed expression of key tumor suppressors or the dysregulated expression of genes governing cell growth and metastasis [64, 65]. The prediction of hypermethylation helps identify potential targets for tumor therapy [66, 67]. Previous study revealed the correlation between DNA methylation patterning and histone modifications, especially H3K4me3, in multiple normal human cells, but not in cancer cells [52]. Another study identified that H3K4me1 pattern at promoter CGI borders in ESCs and normal PrECs could predict DNA methylation encroachment in LNCaP cells [35]. The current study showed that high-level unimodal pattern of H3K4me1 in normal tissues could predict DNA hypermethylation during tumorigenesis in a variety of cancer types. In summary, our study identified a biological benchmark which could pre-mark DNA hypermethylation in cancers.

Bivalent genes, whose promoters are marked by both H3K4me3 and H3K27me3, play critical roles in the differentiation of ESCs [10]. Recently, it is noticed that the role of bivalent genes is not limited to development/differentiation but also cancer progression, such as gliomagenesis, during which the expression of bivalent genes is also regulated by mainly H3K4me3 and H3K27me3 [68]. Our studies revealed the role of a previously unappreciated histone modification-H3K4me1 in the regulation of bivalent genes in the process of development from ESCs to lineage-committed tissues and the development of tumors from normal tissues: our previous study showed that H3K4me1 are accumulated and increased on the promoter of bivalent genes in the process of ESCs differentiation (unpublished data); current study revealed that the hypermethylated genes in cancers are prone to be bivalent genes, moreover, DNA methylation and H3K4me1 showed interplay with each other in the process of carcinogenesis.

DNA methylation and histone modifications are both critical epigenetic marks regulating tumorigenesis, and they can interplay with each other in cancer cells: removal of DNA methylation at promoters leads to increased histone modifications, including H3K4me1/3 and H3K27me3 [54]. Our study furthermore examined this regulation in different groups of genes and identified that DNA methylation ablation differentially regulates the histone modifications between the bivalent and non-bivalent promoters. Bivalent promoters showed significantly more gain of H3K4me1 and H3K27me3 compared with non-bivalent promoters. However, the dynamics of H3K4me1 doesn’t change the DNA methylation level or pattern. There might be an intermediary transitory step in the process of cancer development, and the gain of DNA methylation is premarked by H3K4me1, but not a direct consequence of H3K4me1 modification. Our study dissected the reciprocity between DNA methylation and histone modification in detail, and revealed that DNA methylation status can regulate the histone modifications with preference on bivalent promoters.

LSD1 regulates the expression of bivalent genes via mediating H3K4 methylation through its demethylase activity in human ESCs [34]. Whether it regulates bivalent genes in cancer cells are rarely studied. Current research revealed that LSD1 preferentially modulates bivalent gene expression in colon cancer cells HCT116. The upregulated genes bear significantly increased H3K4 methylation, especially H3K4me1. However, the downregulated genes in LSD1 KO cells don’t show accumulation of H3K4 methylation compared with WT cells. LSD1 is pleiotropic in regulating gene expressions: other than modulating gene expression through modifying the histone methylation, LSD1 can induce gene expression through interacting with nuclear receptors [40]. The downregulated gene expression in LSD1 KO cells is probably due to the lack of LSD1 recruiting nuclear receptors on the promoter, although further experiments are needed to confirm this hypothesis.

LSD1 is previously reported to promote tumor growth and metastasis, however, the underlying mechanisms are not fully understood [41, 69]. Bivalent genes usually promote cells out of stemness into differentiation and thus likely tend to be tumor suppressors. Here, we reported that LSD1 preferentially represses the expression of bivalent genes in cancer cells to promote tumorigenesis, raising a novel mechanism underlying the oncogenic role of LSD1. We confirmed this hypothesis in colon cancer cell HCT116. *OVOL2* is a bivalent gene whose promoter accumulates H3K4me1/3 upon LSD1 knockout in HCT116. Inhibiting *OVOL2* in LSD1 KO cells restored the cancer stem cell phenotype of HCT116 cells. In sum, we proposed a novel oncogenic mechanism of LSD1 and confirm it in HCT116 cells, although more experiments involving more cancer cell types are needed in the future to tamp the foundation for this mechanism.

## Conclusions

In summary, our work systemically explored the data of multiple cancer types and revealed the correlation between H3K4me1 and DNA hypermethylation at the promoter CGIs in cancer cells. Moreover, current research studied the interplay between DNA hypermethylation and H3K4me1 in detail, highlighting a previously unappreciated epigenetic event, reciprocal regulation between H3K4me1 and DNA methylation, in the process of cancer development. The current study also revealed a novel mechanism for the oncogenic role of LSD1. Taken together, this study unveiled a novel mechanism of epigenetic landscape alteration in cancers, providing novel insights into cancer therapy.

## Methods

### Data source

Processed WGBS data of LIHC and normal liver tissue were collected from GSE70090 [43]. Processed WGBS datasets of other tumors (BLCA, BRCA, COAD, LUAD, LUSC, READ, STAD, UCEC) and corresponding normal tissues, as well as GBM were downloaded from [42] https://zwdzwd.github.io/pmd, data of normal brain was downloaded from MethBase [70] (http://smithdata.usc.edu/methbase/data/Lister-Brain-2013/Human_FrontCortexFemale64Yr/tracks_hg19). H3K4me1/3 and H3K27me3 ChIP-seq datasets of normal colon tissue and COAD were collected from GSE136889 [71]. H3K4me1/3 and H3K27me3 ChIP-seq data of other normal tissues used in this study were downloaded from Roadmap (https://egg2.wustl.edu/roadmap/data/byFileType/signal/). The RNA-seq datasets of tumors and tissue normal counterparts used in this study were downloaded from http://duffel.rail.bio/recount/v2/TCGA/rse_gene.Rdata and processed by R package recount 2 [72]. H3K4me1/3 and H3K27me3 ChIP-seq data (GSE58638) and DNA methylation bisulfite sequencing data (GSE58695) of wild type and DNMT1 and DNMT3B double knockout HCT116 cells were collected from the previous study [54]. H3K4me3 and H3K27me3 ChIP-seq datasets of hESCs cell line (H1) were collected from ENCODE (https://www.encodeproject.org/). The H3K4me1/3 ChIP-seq data and RRBS data of LSD1 control and overexpressed NCM460 cells, and the H3K4me1/3 ChIP-seq data, RRBS data and RNA-seq data of HCT116 wild type and LSD1 knockout HCT116 cells of our study were available at NCBI Gene Expression Omnibus (GEO) under accession number GSE222612.

### Gene classification based on promoter CGIs DNA methylation difference between tumors and tissue normal counterparts

The coordinates of CGIs of human genome (hg19) were retrieved using Table Browser (https://genome.ucsc.edu/cgi-bin/hgTables). CGIs overlapped with any TSS of gene transcripts were defined as promoter CGIs and only genes with promoter CGIs were used for the classification analysis. All promoter CGIs were splitted into 20 bins and the DNA methylation level of each bin of CGIs were calculated using the function ‘normalizeToMatrix’ from R package ‘EnrichedHeatmap’. Then based on the distribution of DNA methylation difference between normal tissues and tumors along CGIs, genes with promoter CGIs were classified into 5 groups using the k-means algorithm.

### Tissue-specific gene expression analysis

To evaluate the tissue specificity of genes in each group, Tau values were calculated with tspex (https://github.com/apcamargo/tspex/) based on TPM (transcripts per million) expression data. We downloaded the TPM expression data of representative 10 tissue types from Genotype-Tissue Expression (GTEx) (https://www.gtexportal.org/home/) database. Tissue enrichment analysis was conducted using the R package ‘TissueEnrich’ with default parameters which used Human Protein Atlas (HPA) as reference dataset [47].

### Function enrichment analysis

Enrichment analysis of Gene Ontology (GO) was performed using the R package ‘clusterProfiler’. Only terms with both *p* and *q* values of < 0.05 were considered significantly enriched.

### Cell culture

Human colorectal carcinoma HCT116 cell line was kindly provided by Professor Wenjing Zhao (School of Medicine, Shenzhen Campus of Sun Yat-sen University), and cultured in McCoy’s 5A medium supplemented with 10% fetal bovine serum (FBS) and 100 U/mL penicillin/streptomycin (Procell, CM-0096). Human colonic epithelial cell line NCM460 was purchased from BeNa Culture Collection (Xinyang, China) and cultured in DMEM medium (Gibco, C11965500BT) supplemented with 10% FBS (Lonsera, S712-012S) and 100 U/mL penicillin/streptomycin (Gibco, 15,140,122). HEK 293 T cell lines were kindly provided by Professor Wenxue Zhao (School of Medicine, Shenzhen Campus of Sun Yat-sen University) and cultured in DMEM medium supplemented with 10% FBS and 100 U/mL penicillin/streptomycin. All cell lines were incubated at 37℃ containing 5% CO_2_. All cell lines were authenticated by short tandem repeat (STR) profiling.

### Lentivirus production and infection

LSD1 coding sequence was amplified from the cDNA of HEK 293 T cells and subcloned into the lentiviral vector pCDH (Addgene #72265) for overexpressing LSD1. The primers used for molecular cloning are provided in Supplementary Table S2. GV248 lentivirus vector (GeneChem Co., Ltd., Shanghai, China) was used to stably knock down *OVOL2* gene expression. The targeting shRNA sequences are: sh*OVOL2*: CAGGCATTCGTCCCTACAAAT; negative control (GV-NC): TTCTCCGAACGTGTCACGT. HEK 293 T cells were transfected with pCDH-LSD1/GV248 and packaging plasmids psPAX2 (Addgene #12260) and pMD2.G (Addgene #12259) using Lipofectamine3000 (Thermo Fisher, L3000015) according to the manufacturer's instructions. The supernatant containing viruses was collected, filtered, and used for infection after 24 h and 52 h of transfection. NCM 460 cells or LSD1 KO HCT116 cells were infected with supernatant containing lentivirus mixed with complete culture medium at a ratio of 1:1 for 24 h, and then subjected to 1 μg/ml puromycin treatment for 3 days. Immunoblotting or qPCR was subsequently performed to examine the overexpression or knockdown efficiency.

### Generation of CRISPR knockout clones

The sequence CGCGGAGGCTCTTTCTTGCG in exon 1 of *LSD1* was selected as the CRISPR target as previously described [73] and subcloned into the CRISPR/Cas9 gene editing vector pSpCas9(BB)-2A-Puro (PX459) V2.0 (Addgene, #62988). The CRISPR/Cas9 plasmid was kindly provided by Professor Junjun Ding (Zhongshan school of medicine, Sun Yat-sen University). HCT116 cells were transiently transfected with CRISPR/Cas9 vector via Lipofectamine3000 (Thermo Fisher, L3000015) and then subjected to puromycin (1 μg/mL) (Shanghai yuanye Bio-Technology, R23002) treatment for 24 h for selection. Individual clones were further expanded. The depletion of LSD1 was confirmed by sanger sequencing and immunoblotting.

### Western blot

The cells were collected and lysed at 4 °C with RIPA (Solarbio, R0010) buffer containing protease and phosphatase inhibitors. Protein lysates were resolved by 10% sodium dodecyl sulfate–polyacrylamide gel electrophoresis (SDS–PAGE) and transferred to PVDF membranes (Millipore, ISEQ00010). Membranes were incubated in blocking buffer (5% milk, 0.1% Tween20 in Tris-buffered saline) for 1 h and probed overnight with primary antibodies at 4 °C, followed by incubation with the corresponding HRP-conjugated secondary antibodies. Pierce ECL Plus Western blotting substrate kit (Tanon, 180–5001). The antibodies were used according to the manufacturer’s recommendations were used. Antibodies used for Western blot analyses were as follows: LSD1 (Cell Signaling Technology, 2139S), FLAG (Cell Signaling Technology, #14793), β-actin (Sino Biological, 1,000,166-MM10), Goat anti-rabbit IgG H&L (HRP) (Abcam, ab6721), HRP-conjugated Affinipure Goat Anti-Mouse IgG(H + L) (ProteinTech Group, Inc., SA00001-1).

### Quantitative real-time PCR (qRT-PCR)

Total RNA was extracted with a total RNA extraction kit (Omega Bio-Tek, R6834-01). cDNA was synthesized using RevertAid First Strand cDNA Synthesis Kit (Thermo Fisher Scientific, k1622). Gene expression was detected by real-time qPCR using Sybrgreen detection systems (Vazyme, Q711-02). *Gapdh* was used for qRT-PCR normalization of the samples. All the data were measured in at least three independent experiments. The primer sequences used in this study are shown in Supplementary Table S2.

### Chromatin immunoprecipitation sequencing (ChIP-seq) and ChIP-qPCR

H3K4me1-, H3K4me3- and H3K27me3-ChIP experiments were performed based on the previously described protocol [74, 75]. Briefly, 5 × 10^6^ cells were cross-linked with 1% formaldehyde for 10 min and quenched by 125 mM glycine for 5 min at room temperature with gentle shaking, and washed twice with PBS. Cells were then pelleted and lysed in ice-cold lysis buffer1 (50 mM HEPES–KOH, pH 7.5, 140 mM NaCl, 1 mM EDTA, 10% Glycerol, 0.5% NP-40, 0.25% Triton X-100) and lysis buffer 2 (10 mM Tris–HCl pH 8.0, 200 mM NaCl, 1 mM EDTA, 0.5 mM EGTA) supplemented with cOmplete protease inhibitors (Roche, 4693132001) for 10 min. The cell lysate was sonicated using Covaris M220 with 5% duty factor for 10 min at 4 °C to shear DNA to fragments (200 ~ 1000 bp). Soluble chromatin was diluted in shearing buffer (1 mM EDTA, 10 mM Tris–HCl pH 8.0, 0.1% SDS) with 1% Triton X-100 and 150 mM NaCl and incubated with 3 μg ChIP-grade antibody at 4 °C overnight with gentle shaking. 5% of input was stored prior to the de-crosslinking procedure. 30 μl Protein-G magnetic beads (Thermo Fisher Scientific, 01134323) were used for subsequent pull-down of antibody-chromatin complex by incubating for 2 h at 4 °C with gentle shaking. The beads were washed with the following buffers for 2 times each: IP buffer (0.1% SDS, 1% Triton X-100, 1 mM EDTA, 150 mM NaCl, 10 mM Tris–HCl pH 8.0), High Salt Wash Buffer (0.1% SDS, 1% Triton X-100, 2 mM EDTA, 500 mM NaCl, 20 mM Tris–HCl pH 8.0), LiCl Wash Buffer (250 mM LiCl, 1% NP-40, 2 mM EDTA, 10 mM Tris–HCl pH 8.0), and TE buffer + 50 mM NaCl (1 mM EDTA, 50 mM NaCl, 10 mM Tris–HCl pH 8.0) 1 time. DNA was eluted with Elution Buffer (1% SDS, 10 mM EDTA, 50 mM Tris–HCl pH 8.0) at 65℃ for 30 min with shaking at 1,400 rpm. The supernatant was then collected and incubated at 65 °C for overnight to reverse-crosslink the DNA. The day after, the enriched DNA was treated with 4 µL of 20 mg/mL RNase at 37 °C for 2 h and 4 µL of 20 mg/mL Proteinase K (MIKX, FZ690) at 55 °C for 2 h. The immunoprecipitation DNA was purified by phenol–chloroform-isoamyl alcohol (Solarbio, P1012-100), washed with ethanol, eluted in nuclease-free water, and used for subsequent experiments. The following antibodies were used in ChIP: H3K4me1 (Abcam, ab8895), H3K4me3 (Abcam, ab8580), H3K27me3 (Active motif, 39055), LSD1 (Abcam, ab17721), IgG (Abcam, ab150157). ChIP-seq libraries were constructed and sequenced by the Novogene Bioinformatics Institute (Novogene, Beijing, China). Libraries were sequenced on the Illumina HiSeq 2500 platform.

For ChIP-qPCR, after the immunoprecipitated DNA was purified, qPCR was performed to detect the LSD1 protein binding sites of the DNA samples. The calculation of the ChIP signal is % input = 5% × 2 ^ (CT_input_—CT_sample_). The sequences of the primers were in Supplementary Table S2.

### Reduced representation bisulfite sequencing (RRBS)

Extracted DNA samples were sent to Novogene Bioinformatics Institute (Novogene, Beijing, China) for RRBS. The DNA samples were digested using methylation-insensitive restriction enzyme Mspl. All the cytosines were subjected to methylation-modified sequencing adaptors, DNA fragments with insert lengths within the range of 40–220 base pairs were cut from the gel and bisulfite treatment was carried out using an EZ DNA Methylation Gold Kit (Zymo Research, D5006). PCR amplification was then performed to obtain the final DNA library. After quality checks of the DNA library, the samples were sent for Illumina HiSeq 2500 sequencing.

### RNA sequencing library construction

RNA sequencing libraries were constructed and sequenced by the Novogene Bioinformatics Institute (Novogene, Beijing, China). High-throughput sequencing was performed as paired-end 150 sequencing using a Hiseq 2500 sequencing system.

### Whole genome bisulfite sequencing data processing

For WGBS and RRBS data analysis, raw reads were first trimmed of low-quality reads and adaptor sequences with TrimGalore (version 0.6.6). Then, Bisulfite treated reads were mapped to the hg19 reference genome using Bismark with parameters: “-N 1 –parallel 4 –bowtie2”. Methylation calling of CpG sites was performed using a bismark methylation extractor and merged symmetric CpG sites by coverage2cytosine. The bismark coverage files were imported into R and CpG sites with a minimum of 10 × coverage and were used for further analysis. PMDs and HMDs regions annotations were described in the previous study [42], the PMDs and HMDs definitions annotations were downloaded from https://zwdzwd.github.io/pmd.

### ChIP-seq data analysis

For ChIP-seq data analysis, FastQC (version 0.11.9) was used to access the reads quality of raw data and TrimGalore (version 0.6.6) was used to trim the adaptor and low-quality reads with parameters “-q 25 –phred33 –length 40 -e 0.1 –stringency 3”. After quality control, the remaining reads were mapped to the reference genome hg19 with bowtie2 (version 2.4.5). Only uniquely mapped reads were kept and duplicates were removed by sambamba (version 0.8.2). Then, we call peaks using MACS2 with the following arguments: ‘‘-f BAM -g hs -q 0.05’’ for narrow peaks. To reduce the background noise, overlapping peaks between repetitions were preserved. Histone modification signal tracks for each sample were generated using the deepTools and were normalized to RPKM for visualization in WashU Epigenome Browser.

### RNA-seq data analysis

For RNA-seq data analysis, FastQC (version 0.11.9) was used to access the reads quality of raw data and Trimmomatic (version 0.39) was used to trim the adaptor and low-quality reads. After quality control. The clean read pairs were mapped to the GENCODE reference transcriptomes (GRCh38.p13 v36 release) and quantified with Salmon. Differential gene expression analysis was performed using R package ‘DESeq2’ (version 1.34.0). Differentially expressed genes were defined with *q* value < 0.1, corrected for multiple tests using the Benjamini Hochberg algorithm.

### Wound healing, transwell migration assay and colony formation assay

In brief, HCT116 cells were seeded into 6-well plates. A straight line “wound” was made with a scape using a 10 μL pipette tip. After washing twice with PBS to remove debris, FBS-free medium was added and cells were incubated at 37℃ with 5% CO_2_ for 48 h. Photographs were captured at 0 h and 48 h.

For transwell migration assay, 200 μL of 2.5 × 10^5^/mL HCT116 cell suspension in serum-free medium was added into the up chamber of 8.0 µm transwell chambers (Corning, 353,097) in 24-well plates. 500 μL medium supplemented with 10% FBS was added to the lower chamber. The cells were then incubated for 48 h at 37℃ with 5% CO_2_. Cells that did not migrate through the pores in the upper chambers were gently removed with a cotton swab, while cells that migrated to the underside of the filter were fixed with 4% paraformaldehyde for 30 min and stained with 0.1% crystal violet for 30 min. Subsequently, three random fields of stained cells were photographed by microscope.

For colony formation assay, HCT116 cells were seeded onto 6-well plates at a density of 400 cells per well with McCoy’s 5A medium supplemented with 10% FBS and incubated at 37℃ containing 5% CO_2_ for 12 days. Cell colonies were fixed with 4% paraformaldehyde for 30 min and stained with 0.1% crystal violet for 30 min. Cell colonies (> 50 cells per colony) were counted and photographed.

### Statistical analysis

In this study, each of the experiments was performed with at least two biological replicates unless otherwise specified. Data were presented as the mean ± SD. calculated by GraphPad Prism 6.0 software. Wilcoxon Rank Sum or Student’s *t*-test indicated in each figure was used to calculate *p* values. “ns” represents not significant, *p* ≥ 0.05, “*” represents *p* < 0.05, “**” represents *p* < 0.01, “***” represents *p* < 0.001, “****” represents *p* < 0.0001.

## Supplementary Information

**Additional file 1: Supplementary Figure S1.** Aberrant DNA methylation patterns in different cancers.

**Additional file 2: Supplementary Figure S2.** DNA hypermethylation features of different cancers. A Number of genes in each group in different cancer types. B The overlap of C4 group genes in different cancer types.

**Additional file 3: Supplementary Figure S3.** Gene expression patterns in different cancer types. A Abundance of genes from 5 groups in tumors or tissue normal counterparts. B The distribution of promoter CGIs in 5 groups in PMDs, HMDs and other regions in different cancer types. C The proportion of bivalent and non-bivalent genes in 5 groups in different cancer types. D Tau index of genes in 5 groups in different cancer types.

**Additional file 4: Supplementary Figure S4.** Go analysis of each group of genes in different cancers.

**Additional file 5: Supplementary Figure S5.** Correlations between DNA methylation, histone methylation and gene expression across multiple tissues.

**Additional file 6: Supplementary Figure S6.** Relationship between H3K4me1 and DNA methylation at promoter CGIs. A Distribution pattern and level of H3K4me1 at promoter CGIs and flanked regions in high-H3K4me1 group and low-H3K4me1 group in different normal tissues. B DNA methylation differences (the levels in tumors minus the levels in tissue normal counterparts) in high-H3K4me1 group and low-H3K4me1 group.

**Additional file 7: Supplementary Figure S7.** Generation of LSD1 OE cell line and genome distribution of H3K4me1 peaks of each group. A Expression of FLAG-LSD1 in NCM460 control (NC) and LSD1 OE cells. B Relative mRNA expression of *LSD1* in NCM460 control (NC) and LSD1 OE cells. C The overlap of H3K4me1 peaks in NC and LSD1 OE NCM460 cells. D Genomic distribution of H3K4me1 peaks of each group.

**Additional file 8: Supplementary Figure S8.** Generation of LSD1 KO cell lines and enrichment of H3K27me3 at promoter CGIs. A Expression of LSD1 in WT and LSD1 KO HCT116 cells. B Enrichment of H3K27me3 at promoter CGIs in WT and LSD1 KO cells.

**Additional file 9: Supplementary Figure S9.** The role of LSD1 in regulating multiple epigenetic modifications. A Genome distribution of H3K4me1 peaks and H3K4me3 peaks. B, C DNA methylation and enrichment of H3K4me1/3 at promoters of Up genes and Down genes in WT and LSD1 KO cells.

**Additional file 10: Supplementary Figure S10.** Enrichment of H3K4me3 and H3K27me3 at promoter of *OVOL2* in H1 cells.

**Additional file 11: Supplementary Figure S11.** Generation of *LSD1* OE and *OVOL2* KD cell lines. A Relative mRNA expression of *LSD1 *and *OVOL2* in control (NC) and *LSD1* OE HCT116 cells. B Relative mRNA expression of *OVOL2* in control (GV-NC) and *OVOL2 *KD cells.

**Additional file 12: Supplementary Figure S12.** Uncropped blots used in this study.

**Additional file 13: Supplementary Table S1.** Summary of 5 gene groups in different cancer types.

**Additional file 14: Supplementary Table S2.** Primer information used in this study.

**Additional file 15: Supplementary Table S3.** Public datasets used in this study.

## Data Availability

The data used in this study are presented in this study and supplementary materials. The datasets generated for this study are available in the NCBI Gene Expression Omnibus (GEO) under accession number GSE222612. The information of public datasets used in this study are provided in the Supplementary Table S3.

## Abbreviations

CGIs: CpG islands
OVOL2: Ovo like zinc finger 2
ESCs: Embryonic stem cells
COMPASS: Complex of Proteins Associated with Set1
HDM: Histone demethylase
PrECs: Prostate epithelial cells
WGBS: Whole-genome bisulfite sequencing
ChIP-seq: Chromatin immunoprecipitation sequencing
RRBS: Reduced representation bisulfite sequencing
RNA-seq: RNA sequencing
GO: Gene Ontology
BLCA: Bladder urothelial carcinoma
BRCA: Breast invasive carcinoma
COAD: Colon adenocarcinoma
LUAD: Lung adenocarcinoma
LUSC: Lung squamous cell carcinoma
READ: Rectum adenocarcinoma
STAD: Stomach adenocarcinoma
UCEC: Uterine corpus endometrial carcinoma
GBM: Glioblastoma multiforme
LIHC: Liver hepatocellular carcinoma
PMDs: Partially methylated domains
HMDs: Highly methylated domains
GTEx: Genotype-Tissue Expression project
HPA: Human Protein Atlas
TSS: Transcription start site
IGV: Integrative Genomics Viewer
