# Supplementary material for: Pan-cancer analysis revealed H3K4me1 at bivalent promoters premarks DNA hypermethylation during tumor development and identified the regulatory role of DNA methylation in relation to histone modifications
Source: BMC Genomics. 2023 May 4;24:235. doi: 10.1186/s12864-023-09341-1 (PMC10157937; doi:10.1186/s12864-023-09341-1)
Supplement: Supplementary file 1 — Additional file 1: Supplementary Figure S1. Aberrant DNA methylation patterns in different cancers. [file 12864_2023_9341_MOESM1_ESM.pdf]

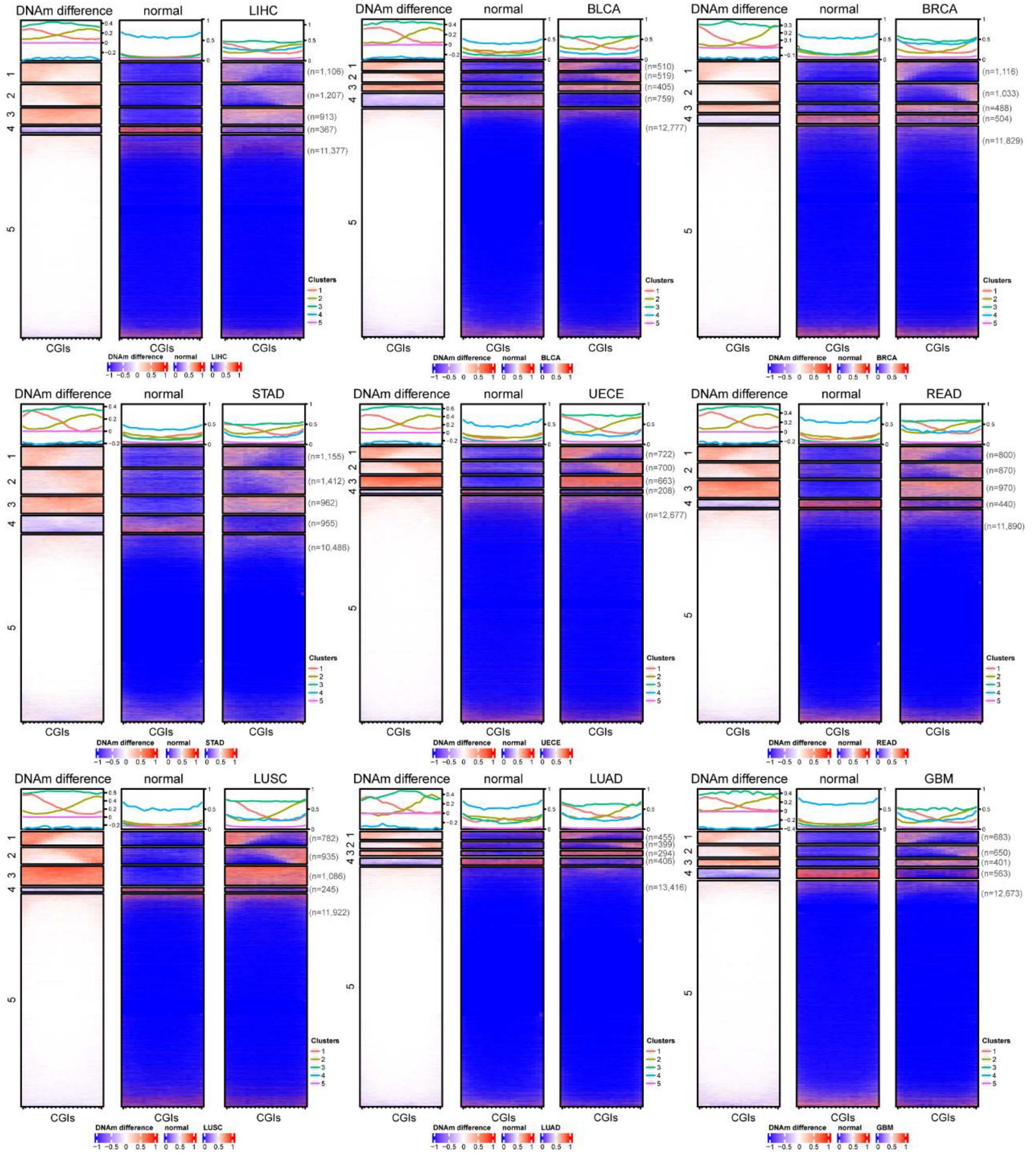

Supplementary Figure S1. Aberrant DNA methylation patterns in different cancers. DNA methylation patterns at promoter CGIs in normal tissues (middle panel) and corresponding cancers (right panel), and DNA methylation difference at promoter CGIs between cancers and tissue normal counterparts (left panel). Each line represents a single CGI.
