# Supplementary material for: Pan-cancer analysis revealed H3K4me1 at bivalent promoters premarks DNA hypermethylation during tumor development and identified the regulatory role of DNA methylation in relation to histone modifications
Source: BMC Genomics. 2023 May 4;24:235. doi: 10.1186/s12864-023-09341-1 (PMC10157937; doi:10.1186/s12864-023-09341-1)
Supplement: Supplementary file 2 — Additional file 2: Supplementary Figure S2. DNA hypermethylation features of different cancers. A Number of genes in each group in different cancer types. B The overlap of C4 group genes in different cancer types. [file 12864_2023_9341_MOESM2_ESM.pdf]

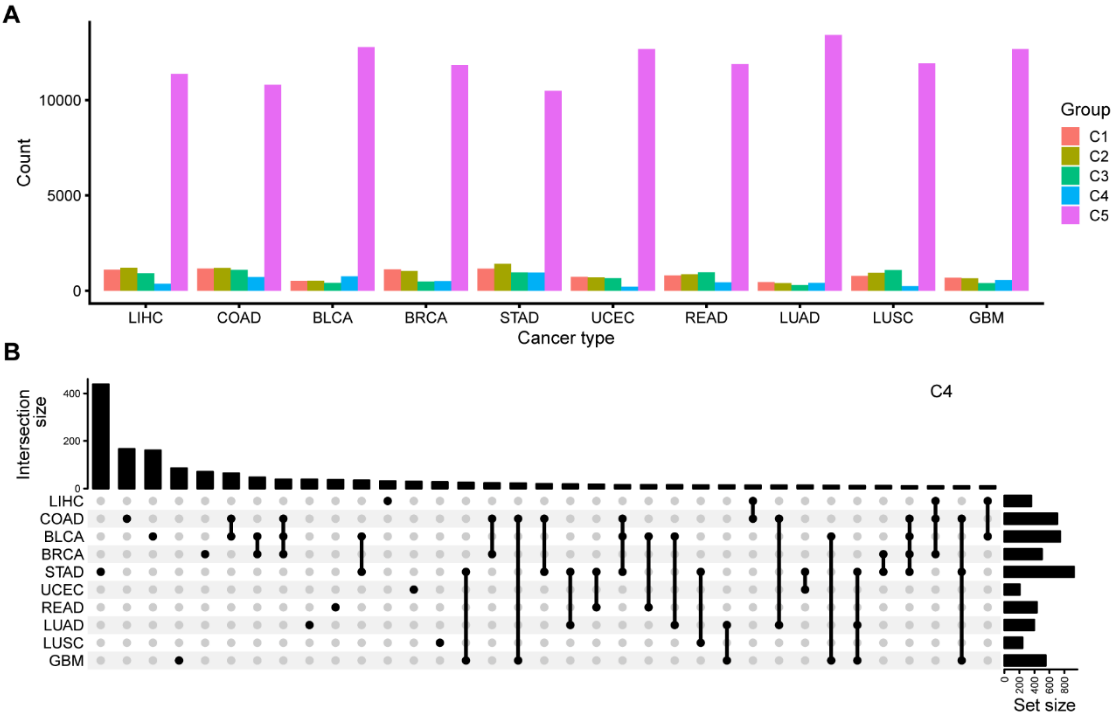

Supplementary Figure S2. DNA hypermethylation features of different cancers. **A** Number of genes in each group in different cancer types. **B** Upset plot showing the overlap of C4 group genes in different cancer types.
