# Supplementary material for: Pan-cancer analysis revealed H3K4me1 at bivalent promoters premarks DNA hypermethylation during tumor development and identified the regulatory role of DNA methylation in relation to histone modifications
Source: BMC Genomics. 2023 May 4;24:235. doi: 10.1186/s12864-023-09341-1 (PMC10157937; doi:10.1186/s12864-023-09341-1)
Supplement: Supplementary file 3 — Additional file 3: Supplementary Figure S3. Gene expression patterns in different cancer types. A Abundance of genes from 5 groups in tumors or tissue normal counterparts. B The distribution of promoter CGIs in 5 groups in PMDs, HMDs and other regions in different cancer types. C The proportion of bivalent and non-bivalent genes in 5 groups in different cancer types. D Tau index of genes in 5 groups in different cancer types. [file 12864_2023_9341_MOESM3_ESM.pdf]

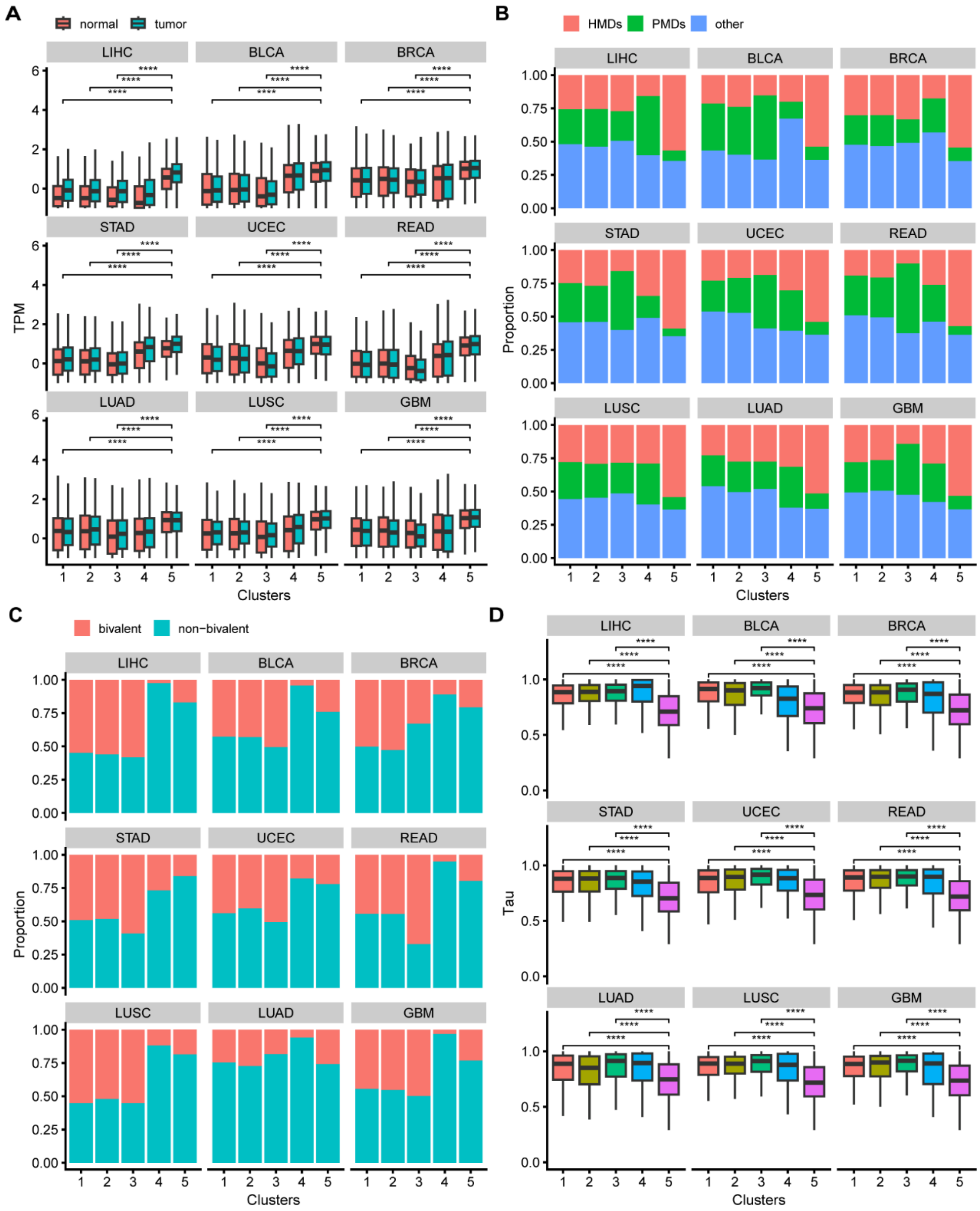

Supplementary Figure S3. Gene expression patterns in different cancer types. **A** Box plots showing abundance of genes from 5 groups tumors or tissue normal counterparts. TPM: transcripts per million. Data were presented as mean± SD. Statistical analysis was performed by Wilcoxon Rank Sum, ****p< 0.0001. **B** The stacked bar plots showing distribution of promoter CGIs in 5 groups in PMDs, HMDs and other regions in different cancer types. **C** The stacked bar plots showing proportion of bivalent and non-bivalent genes in 5 groups in different cancer types. **D** Box plots showing Tau index of genes in 5 groups in different cancer types. Data were presented as mean ± SD. Statistical analysis was performed by Wilcoxon Rank Sum, **** p < 0.0001.
