# Supplementary material for: Pan-cancer analysis revealed H3K4me1 at bivalent promoters premarks DNA hypermethylation during tumor development and identified the regulatory role of DNA methylation in relation to histone modifications
Source: BMC Genomics. 2023 May 4;24:235. doi: 10.1186/s12864-023-09341-1 (PMC10157937; doi:10.1186/s12864-023-09341-1)
Supplement: Supplementary file 4 — Additional file 4: Supplementary Figure S4. Go analysis of each group of genes in different cancers. [file 12864_2023_9341_MOESM4_ESM.pdf]

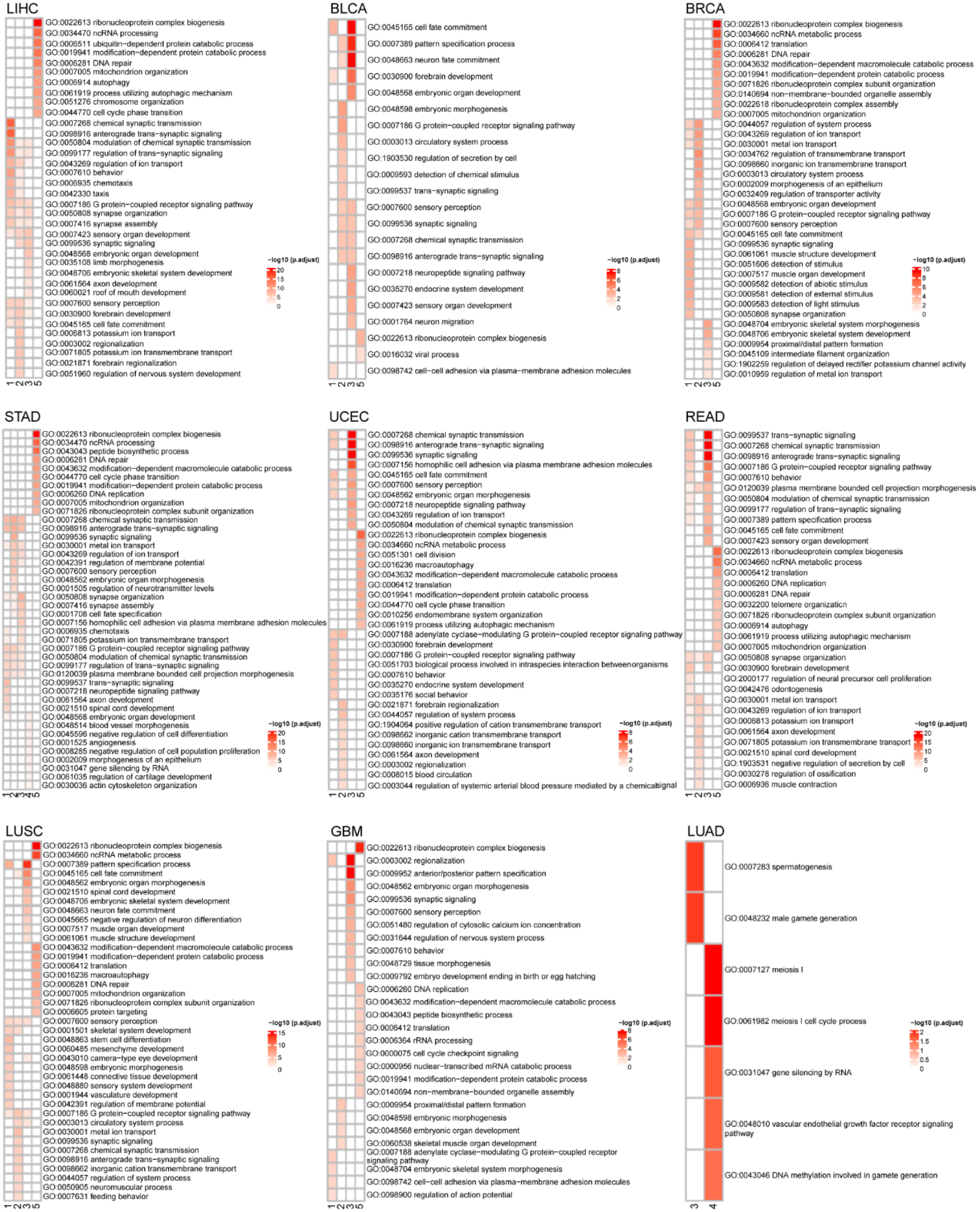

Supplementary Figure S4. Functional characterization of genes in different cancers. Go analysis of each group of genes in different cancers.
