# Supplementary material for: Pan-cancer analysis revealed H3K4me1 at bivalent promoters premarks DNA hypermethylation during tumor development and identified the regulatory role of DNA methylation in relation to histone modifications
Source: BMC Genomics. 2023 May 4;24:235. doi: 10.1186/s12864-023-09341-1 (PMC10157937; doi:10.1186/s12864-023-09341-1)
Supplement: Supplementary file 5 — Additional file 5: Supplementary Figure S5. Correlations between DNA methylation, histone methylation and gene expression across multiple tissues. [file 12864_2023_9341_MOESM5_ESM.pdf]

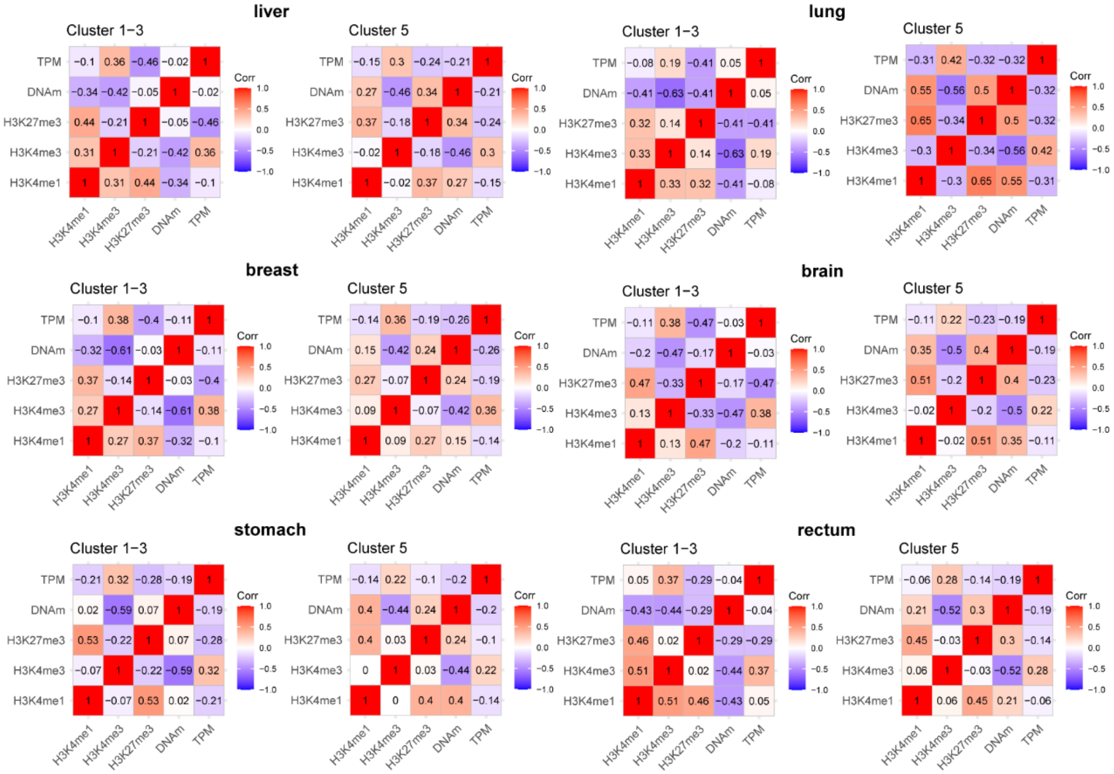

Supplementary Figure S5. Correlations between DNA methylation, histone methylation and gene expression across multiple tissues. Heatmaps showing pairwise spearman correlation coefficients among multiple epigenetic modifications and gene expression. DNAm, DNA methylation. TPM: transcripts per million.
