# Supplementary material for: Pan-cancer analysis revealed H3K4me1 at bivalent promoters premarks DNA hypermethylation during tumor development and identified the regulatory role of DNA methylation in relation to histone modifications
Source: BMC Genomics. 2023 May 4;24:235. doi: 10.1186/s12864-023-09341-1 (PMC10157937; doi:10.1186/s12864-023-09341-1)
Supplement: Supplementary file 6 — Additional file 6: Supplementary Figure S6. Relationship between H3K4me1 and DNA methylation at promoter CGIs. A Distribution pattern and level of H3K4me1 at promoter CGIs and flanked regions in high-H3K4me1 group and low-H3K4me1 group in different normal tissues. B DNA methylation differences (the levels in tumors minus the levels in tissue normal counterparts) in high-H3K4me1 group and low-H3K4me1 group. [file 12864_2023_9341_MOESM6_ESM.pdf]

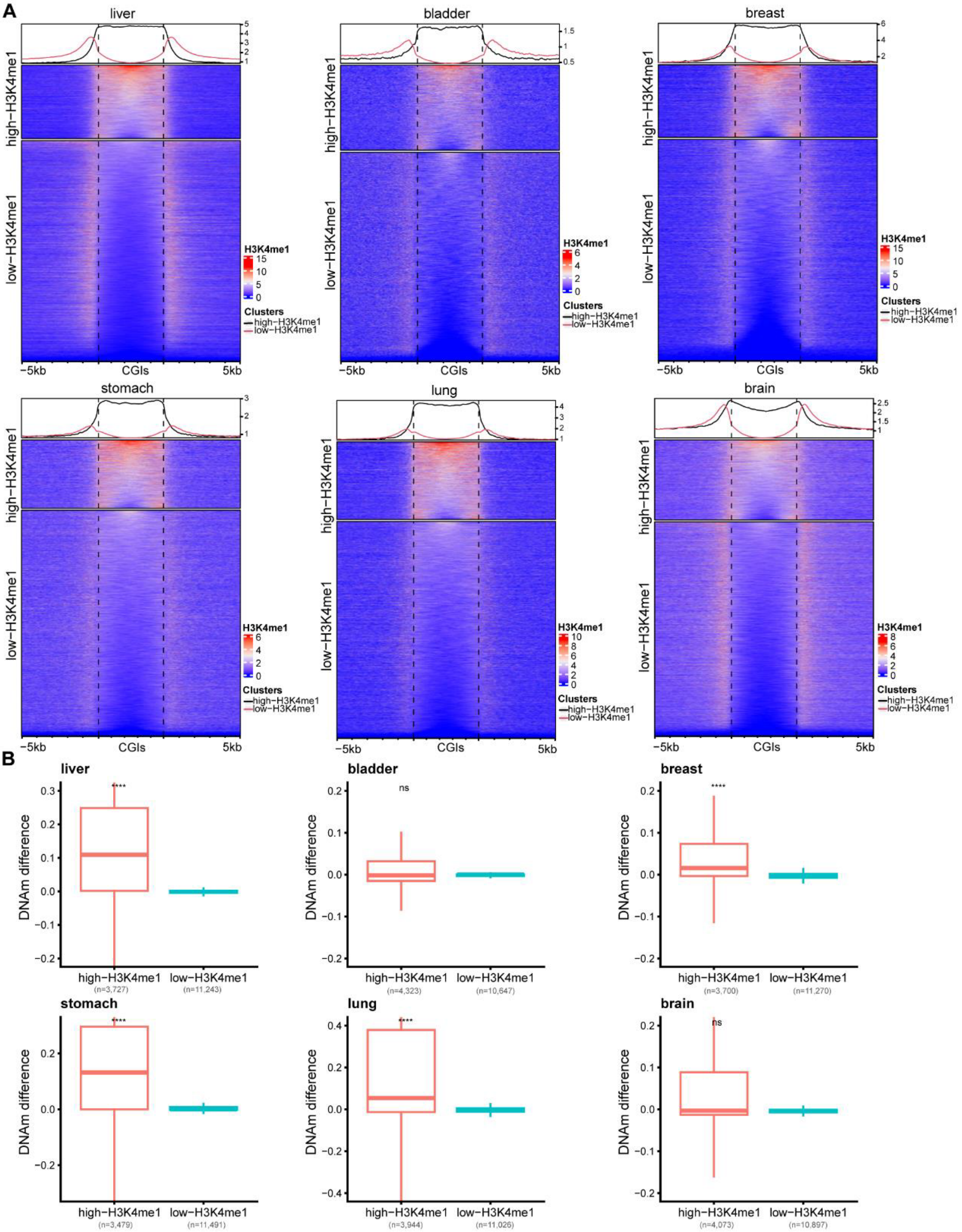

Supplementary Figure S6. Relationship between H3K4me1 and DNA methylation at promoter CGIs. **A** Distribution pattern and level of H3K4me1 at promoter CGIs and flanked regions in high-H3K4me1 group and low-H3K4me1 group in different normal tissues. **B** The box plots showing DNA methylation differences (the levels in tumors minus the levels in tissue normal counterparts) in high-H3K4me1 group and low-H3K4me1 group. DNAm, DNA methylation. Data were presented as mean± SD. Statistical analysis was performed by Wilcoxon Rank Sum, ns: not significantly, *****p*< 0.0001.
