# Supplementary material for: Pan-cancer analysis revealed H3K4me1 at bivalent promoters premarks DNA hypermethylation during tumor development and identified the regulatory role of DNA methylation in relation to histone modifications
Source: BMC Genomics. 2023 May 4;24:235. doi: 10.1186/s12864-023-09341-1 (PMC10157937; doi:10.1186/s12864-023-09341-1)
Supplement: Supplementary file 7 — Additional file 7: Supplementary Figure S7. Generation of LSD1 OE cell line and genome distribution of H3K4me1 peaks of each group. A Expression of FLAG-LSD1 in NCM460 control (NC) and LSD1 OE cells. B Relative mRNA expression of LSD1 in NCM460 control (NC) and LSD1 OE cells. C The overlap of H3K4me1 peaks in NC and LSD1 OE NCM460 cells. D Genomic distribution of H3K4me1 peaks of each group. [file 12864_2023_9341_MOESM7_ESM.pdf]

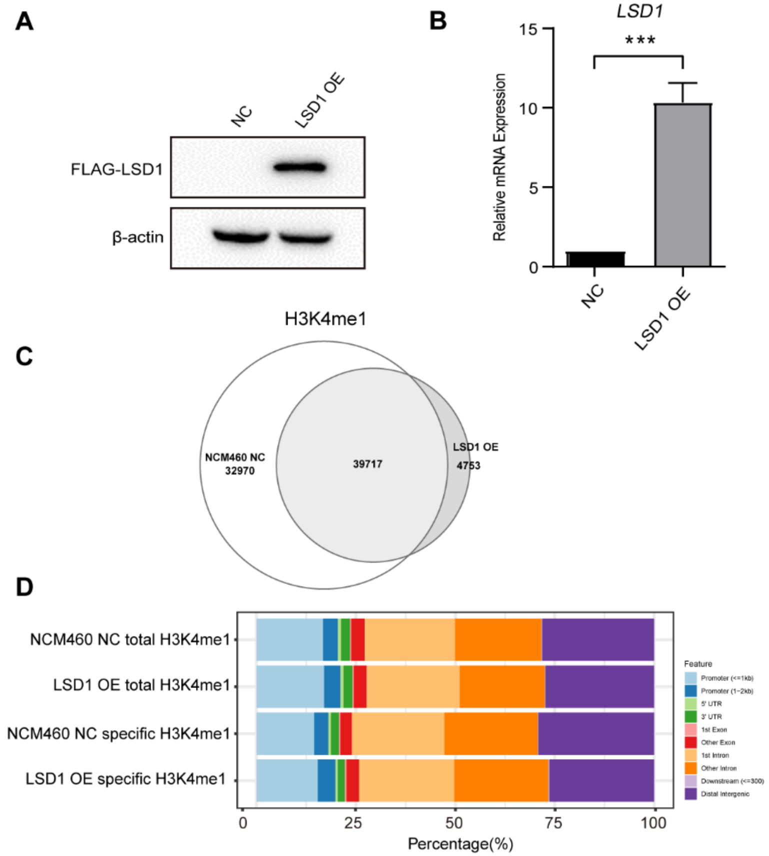

Supplementary Figure S7. Generation of LSD1 OE cell line and genome distribution of H3K4me1 peaks of each group. **A** Expression of FLAG-LSD1 in NCM460 control (NC) and LSD1 OE cells, β-actin was set as loading control. The grouping of blots cropped from different parts of the same gel was divided with white space. Uncropped full-length blots are presented in Supplementary Figure S12. **B** Relative mRNA expression of *LSD1* in NCM460 control (NC) and LSD1 OE cells. Data were presented as mean± SD. Statistical analysis was performed by Student's t test. $***p < 0.001$. **C** The overlap of H3K4me1 peaks in NC and LSD1 OE NCM460 cells. **D** The stacked bar plots showing genomic distribution of H3K4me1 peaks of each group. NCM460 NC total H3K4me1: total genes with H3K4me1peaks in NCM460 cells; LSD1 OE total H3K4me1: total genes with H3K4me1 peaks in LSD1 OE cells; NCM460 NC specific H3K4me1: group of genes with H3K4me1 peaks only in NCM460 NC cells; LSD1 OE specific H3K4me1 peaks: group of genes with H3K4me1 peaks only in LSD1 OE cells.
