# Supplementary material for: Pan-cancer analysis revealed H3K4me1 at bivalent promoters premarks DNA hypermethylation during tumor development and identified the regulatory role of DNA methylation in relation to histone modifications
Source: BMC Genomics. 2023 May 4;24:235. doi: 10.1186/s12864-023-09341-1 (PMC10157937; doi:10.1186/s12864-023-09341-1)
Supplement: Supplementary file 8 — Additional file 8: Supplementary Figure S8. Generation of LSD1 KO cell lines and enrichment of H3K27me3 at promoter CGIs. A Expression of LSD1 in WT and LSD1 KO HCT116 cells. B Enrichment of H3K27me3 at promoter CGIs in WT and LSD1 KO cells. [file 12864_2023_9341_MOESM8_ESM.pdf]

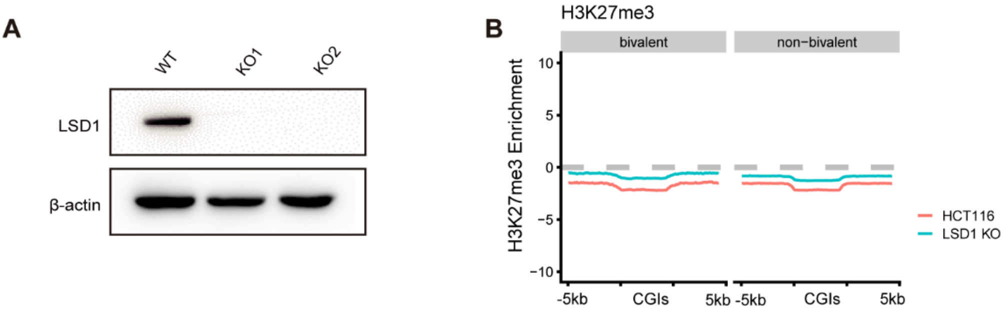

Supplementary Figure S8. Generation of LSD1 KO cell lines and enrichment of H3K27me3 at promoter CGIs. **A** Expression of LSD1 in WT and LSD1 KO HCT116 cells, β-actin was set as loading control. The grouping of blots cropped from different parts of the same gel was divided with white space. Uncropped full-length blots are presented in Supplementary Figure S12. **B** Enrichment of H3K27me3 at promoter CGIs in WT and LSD1 KO HCT116 cells.
