# Supplementary material for: Pan-cancer analysis revealed H3K4me1 at bivalent promoters premarks DNA hypermethylation during tumor development and identified the regulatory role of DNA methylation in relation to histone modifications
Source: BMC Genomics. 2023 May 4;24:235. doi: 10.1186/s12864-023-09341-1 (PMC10157937; doi:10.1186/s12864-023-09341-1)
Supplement: Supplementary file 9 — Additional file 9: Supplementary Figure S9. The role of LSD1 in regulating multiple epigenetic modifications. A Genome distribution of H3K4me1 peaks and H3K4me3 peaks. B, C DNA methylation and enrichment of H3K4me1/3 at promoters of Up genes and Down genes in WT and LSD1 KO cells. [file 12864_2023_9341_MOESM9_ESM.pdf]

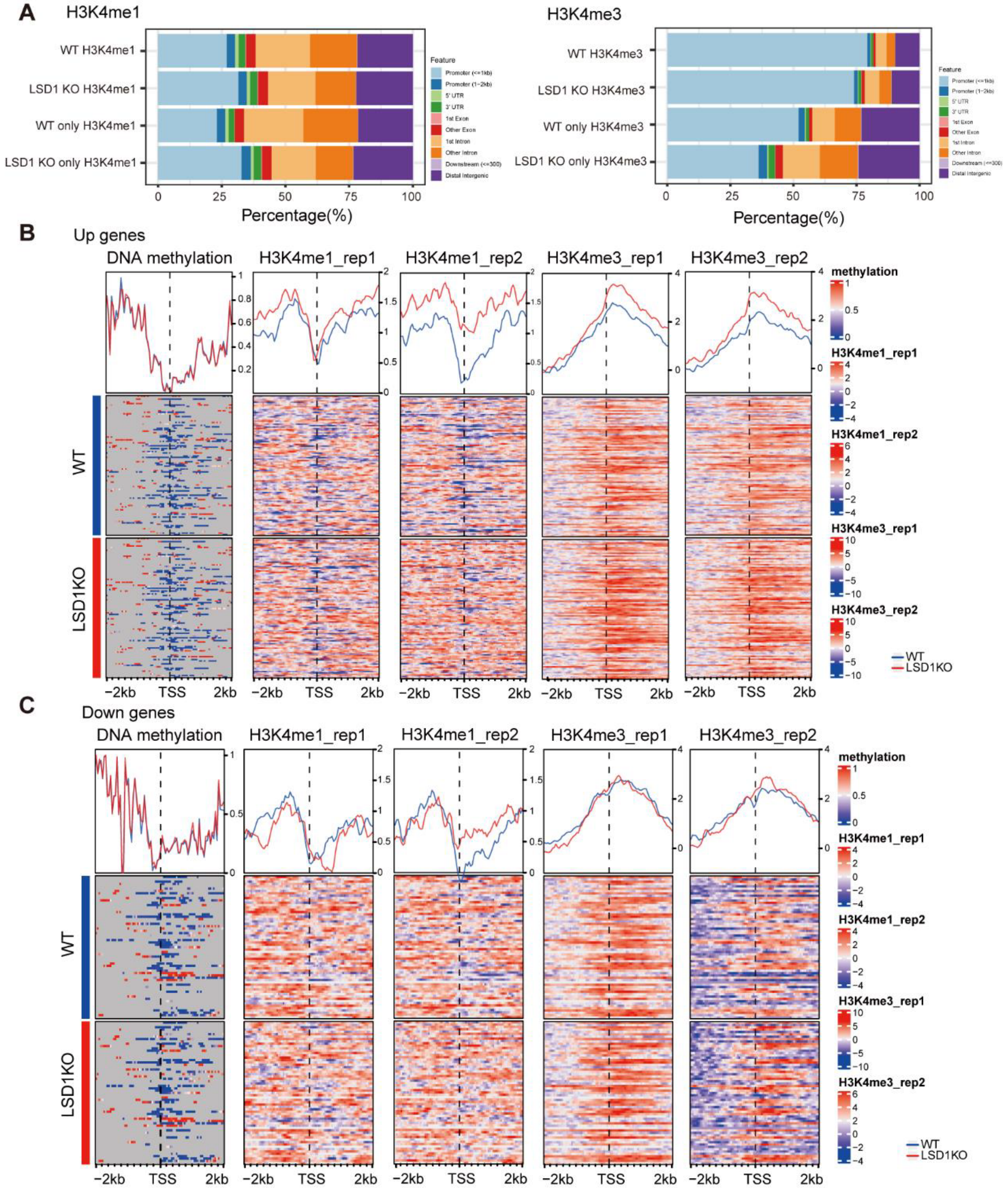

Supplementary Figure S9. The role of LSD1 in regulating multiple epigenetic modifications. **A** Genome distribution of H3K4me1 peaks (left panel) and H3K4me3 peaks (right panel). Left panel, WT H3K4me1: total genes with H3K4me1 peaks in WT HCT116 cells; LSD1 KO H3K4me1: total genes with H3K4me1 peaks in LSD1 KO cells; WT only H3K4me1 peaks: group of genes with H3K4me1 peaks only in WT HCT116 cells; LSD1 KO only H3K4me1: group of genes with H3K4me1 peaks only in LSD1 KO cells. Right panel, WT H3K4me3: total genes with H3K4me3 peaks in WT HCT116 cells; LSD1 KO H3K4me3: total genes with H3K4me3 peaks in LSD1 KO cells; WT only H3K4me3: group of genes with H3K4me3 peaks only in WT HCT116 cells; LSD1 KO only H3K4me3: group of genes with H3K4me3 peaks only in LSD1 KO cells. **B, C** DNA methylation and enrichment of H3K4me1/3 at promoters of Up genes and Down genes in WT and LSD1 KO cells. Up genes, genes that are upregulated in LSD1 KO cells compared with WT cells. Down genes, genes that are downregulated in LSD1 KO cells compared with WT cells.
