# Supplementary figures and images for: Pan-cancer analysis revealed H3K4me1 at bivalent promoters premarks DNA hypermethylation during tumor development and identified the regulatory role of DNA methylation in relation to histone modifications
Source: BMC Genomics. 2023 May 4;24:235. doi: 10.1186/s12864-023-09341-1 (PMC10157937; doi:10.1186/s12864-023-09341-1)

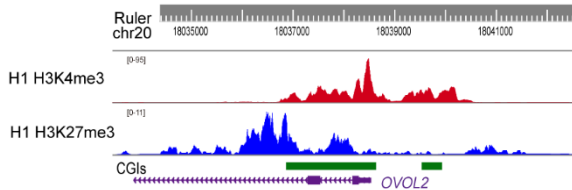

**Supplementary Figure S10.** Enrichment of H3K4me3 and H3K27me3 at promoter of *OVOL2* in H1 cells.

Supplement: Supplementary file 10 — Additional file 10: Supplementary Figure S10. Enrichment of H3K4me3 and H3K27me3 at promoter of OVOL2 in H1 cells. [file 12864_2023_9341_MOESM10_ESM.pdf]
