# Supplementary material for: Pan-cancer analysis revealed H3K4me1 at bivalent promoters premarks DNA hypermethylation during tumor development and identified the regulatory role of DNA methylation in relation to histone modifications
Source: BMC Genomics. 2023 May 4;24:235. doi: 10.1186/s12864-023-09341-1 (PMC10157937; doi:10.1186/s12864-023-09341-1)
Supplement: Supplementary file 11 — Additional file 11: Supplementary Figure S11. Generation of LSD1 OE and OVOL2 KD cell lines. A Relative mRNA expression of LSD1 and OVOL2 in control (NC) and LSD1 OE HCT116 cells. B Relative mRNA expression of OVOL2 in control (GV-NC) and OVOL2 KD cells. [file 12864_2023_9341_MOESM11_ESM.pdf]

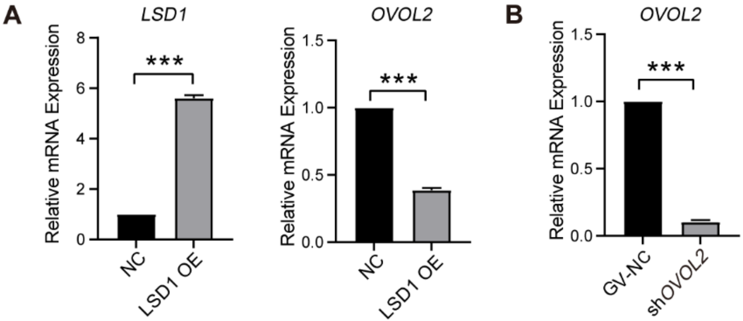

Supplementary Figure S11. Generation of *LSD1* OE and *OVOL2* KD cell lines. **A** Relative mRNA expression of *LSD1* (left panel) and *OVOL2* (right panel) in control (NC) and *LSD1* OE HCT116 cells. Data were presented as mean± SD. Statistical analysis was performed by Student's *t* test, ***$p < 0.001$. **B** Relative mRNA expression of *OVOL2* in control (GV-NC) and *OVOL2* KD cells. Data were presented as mean± SD. Statistical analysis was performed by Student's *t* test, ***$p < 0.001$.
