# Supplementary material for: Pan-cancer analysis revealed H3K4me1 at bivalent promoters premarks DNA hypermethylation during tumor development and identified the regulatory role of DNA methylation in relation to histone modifications
Source: BMC Genomics. 2023 May 4;24:235. doi: 10.1186/s12864-023-09341-1 (PMC10157937; doi:10.1186/s12864-023-09341-1)
Supplement: Supplementary file 12 — Additional file 12: Supplementary Figure S12. Uncropped blots used in this study. [file 12864_2023_9341_MOESM12_ESM.pdf]

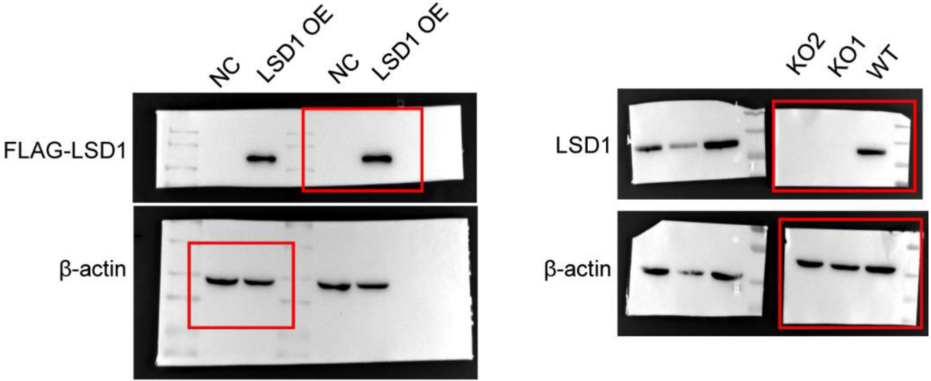

Supplementary Figure S12. Uncropped blots used in this study. Uncropped blots related to Supplementary Figure S7A (left panels) and Supplementary Figure S8A (right panels). The red frame indicated uncropped blots used in this study.
